# Activation of Wnt/β-Catenin Signaling Involves 660 nm Laser Radiation on Epithelium and Modulates Lipid Metabolism

**DOI:** 10.3390/biom12101389

**Published:** 2022-09-29

**Authors:** Qiyang Xiao, Lijing Wang, Juling Zhang, Xinyu Zhong, Zhou Guo, Jiahao Yu, Yuanyuan Ma, Haigang Wu

**Affiliations:** 1 School of Artificial Intelligence, Henan University, Zhengzhou 450046, China; 2 School of Life Sciences, Henan University, Kaifeng 475000, China; 3 Center for Faculty Development, South China Normal University, Guangzhou 510631, China; 4 Shandong Zhongbaokang Medical Implements Co., Ltd., Zibo 255000, China; 5 School of Pharmacy, Henan University, Kaifeng 475000, China

**Keywords:** breast, RNA sequencing, metabolite, purine metabolism, Wnt/β-catenin

## Abstract

Research has proven that light treatment, specifically red light radiation, can provide more clinical benefits to human health. Our investigation was firstly conducted to characterize the tissue morphology of mouse breast post 660 nm laser radiation with low power and long-term exposure. RNA sequencing results revealed that light exposure with a higher intervention dosage could cause a number of differentially expressed genes compared with a low intervention dosage. Gene ontology analysis, protein–protein interaction network analysis, and gene set enrichment analysis results suggested that 660 nm light exposure can activate more transcription-related pathways in HC11 breast epithelial cells, and these pathways may involve modulating critical gene expression. To consider the critical role of the Wnt/T-catenin pathway in light-induced modulation, we hypothesized that this pathway might play a major role in response to 660 nm light exposure. To validate our hypothesis, we conducted qRT-PCR, immunofluorescence staining, and Western blot assays, and relative results corroborated that laser radiation could promote expression levels of β-catenin and relative phosphorylation. Significant changes in metabolites and pathway analysis revealed that 660 nm laser could affect nucleotide metabolism by regulating purine metabolism. These findings suggest that the Wnt/β-catenin pathway may be the major sensor for 660 nm laser radiation, and it may be helpful to rescue drawbacks or side effects of 660 nm light exposure through relative interventional agents.

## 1. Introduction

Light therapy, utilizing laser or light-emitting diodes (LEDs), has been widely used for pursuing clinical benefits [1,2]. Light with a wavelength ranging between 600 nm and 900 nm has been proven to be involved in affecting cellular biology processes and has been further applied to disease therapy, for example, acne vulgaris therapy [3], photodynamic therapy [4], corneal attenuating endothelial cell dysfunction [5], and delaying sleep–wake phase disorder [6].

A 660 nm laser displaying a lower permeability of animal tissues [7] has been widely used for skin disease therapy, including modulating dentinogenesis and angiogenesis in vitro [8], promoting the expression levels of VEGF and CD34 [9], and skin wound healing [10,11,12]. These biological effects of light radiation are decided by the intervention dosage, strategies, and targeted organisms. Based on the different approaches to therapeutic interventions, biological effects and molecular mechanisms of 660 nm laser radiation on tissue are often different, for example, modulating autophagy to ensure hemostasis [13], modulating MMP-2 expression to promote angiogenic processes [14], inhibiting the FOXO1 signaling pathway to reduce oxidative stress [15], and downregulating NF-κB transcriptional activity with higher cAMP to induce inflammation [16]. Owing to its excellent features, LED light or laser has been widely used in breast surgery, for example, carbon dioxide for breast lumpectomy [17], breast ptosis surgery [18], and 3D laser imaging [19]. A major risk of light radiation to the breast may be as a contributor to breast cancer, which is the most dangerous cancer for females (4,055,770 diagnosed per year in the USA) [20]. Studies have proven that light can strongly interact with estrogen or related membrane receptors, for example, photoreactivity [21], estrogen–DNA adducts, or DNA damage [22]. To date, few studies have focused on the investigation of a 660 nm laser on breast tissue. Consequently, to determine how 660 nm laser exposure affects molecular regulation networks, it is necessary to investigate the biological effects of a 660 nm laser on breast tissue, especially for epithelial cells.

Here, we conducted H&E staining to examine the morphological changes of breast tissue post 660 nm laser radiation, providing global insights into the influence of laser radiation. Then, RNA sequencing of breast epithelial cells (HC11) post 660 nm laser radiation with two different intervention dosages was conducted. In addition, bioinformatics analysis of the gene profile was employed to identify the critical pathways modulated by laser radiation, including gene ontology (GO) analysis, protein–protein interaction (PPI) network analysis, and gene set enrichment analysis (GSEA). Metabolome changes in HC11 cells post intervention were investigated using liquid chromatography–mass spectrometry (LC–MS), and enrichment pathway analysis of significantly changed metabolites was conducted using the MetaboAnalyst database. Subsequently, qRT-PCR, immunofluorescence staining, and Western blot analysis were conducted to validate whether critical pathways were activated or not. Our investigation provides greater insights into the biological effects of 660 nm laser radiation, and it may guide laser radiation therapy for breast-related diseases.

## 2. Materials and Methods

**Animal experiment:** BALB/c mice (*n* = 3, female) were anesthetized using chloral hydrate, and fur of breast part was removed to expose total breast tissue. Then, mouse was fixed using adapter and exposed to 660 nm laser (power 10 mW, total intervention dosage 10 J/cm^2^). Breast tissue was obtained and fixed in 4% polyformaldehyde for 24 h. The fixed tissue was delivered to Servicebio company (Beijing, China) for further H&E staining.

**Cell culture:** Mouse normal breast cell line HC11 was purchased from the American Type Culture Collection (ATCC, Manassas, VA, USA). Cells were cultured in complete medium, RPMI 1640 (Gibco, ThermoFisher Scientific, Shanghai, China, Cat. 11875093) containing L-glutamine, 50 μg/mL gentamycin, 10% Fetal Bovine Serum (FBS, Cat. 10270-106, Gibco, ThermoFisher Scientific, Shanghai, China,), 10 ng/mL EGF (Beyotime, shanghai, China, P6114-100 μg), 5 μg/mL insulin (Cat. C600366, Sangon Biotech, China), and 100-unit penicillin/streptomycin (Cat. 15140122, Gibco, ThermoFisher Scientific, Shanghai, China). The culturing environment was set as 5% CO_2_ at 37 °C.

**RNA sequencing**: HC11 cells in 12-well dish were exposed to 660 nm laser (10 mW, 0.5 J/cm^2^ and 1 J/cm^2^). Then, total RNA of HC11 cells was extracted with Total RNA Extraction Kit (Solarbio, Shanghai, China). These RNA samples (*n* = 3 per group) were stored at −80 °C and then delivered to Novogene company for further RNA sequencing process (HiSeq 2500). The raw data of RNA sequencing were uploaded to GEO database (GSE207183).

**Bioinformatics analysis**: RNA sequence libraries were generated with standard mRNA stranded protocols from Illumina and sequenced on a Hiseq. 2500 (pair-end reads 150 bp long, RapidRun mode) at the Novogene company, China. Data processing was carried out on personal computer (Intel i5 CPU). The generated reads were mapped to the mouse genome version GRCm39 using Tophat v.2.1.1. Read data were converted to gene counts with the program htseq v.1.99.2. Differential gene expression was assessed using Bioconductor DESeq. 3.0 package [23] running in R language version 4.1.2. Only genes with *p*-values lower than 0.05 after FDR correction for multiple testing were considered differentially expressed genes.

Volcano plot and heatmap of differentially expressed genes were created using ggplot2 (30 December 2016) running in Rstudio (Version 1.4.1717). Protein–protein interaction (PPI) network was obtained from STRING website (https://cn.string-db.org/, 1 March 2022, organism mus musculus, and minimum required interaction score—high confidence), and images of PPI network were produced using Cytoscape 3.8.2. Gene ontology (GO) analysis of DEGs was conducted in g:profiler website (https://biit.cs.ut.ee/gprofiler/gost, 1 March 2022,) with ordered query. The bubble plot of GO analysis was generated using ggplot2 running in Rstudio. Gene score enrichment analysis (GSEA) was conducted in GSEA software v.4.2.2 (http://www.gsea-msigdb.org/gsea/index.jsp, 1 March 2022,) with Hallmark gene set, and parameters were set as following: number of permutations, 3000; permutation type, gene set. Gene overlapping was conducted in FunRich3.1.3. Permutation types of GSEA were set as “gene set”, and other parameters were set as default if without any special mention.

**Immunofluorescence staining:** For immunofluorescence staining, cells post treatment (*n* = 3 replicates per group) were fixed in 4% polyformaldehyde for 30 min, and fixed cells were incubated with primary antibody of rabbit anti-β-catenin (Abcam, Shanghai, China, ab32572, conjugated with Alexa Fluor 647 dye) at 4 °C overnight. Fixed cells were counterstained with DAPI to display nuclei. Photos were obtained by a fluorescent microscope with a camera (Zeiss LSM 880, Germany). Excitation and emission wavelengths were 405 and 425–460 nm (blue) for DAPI and 652 and 668 nm (red) for Alexa Fluor 647.

**Western blot**: Cultured HC11 cells (*n* = 3 replicates per group) were lysed in radioimmunoprecipitation (RIPA, Roo30, Solarbio, Beijing, China) buffer containing PMSF, and total proteins were measured using the BCA Protein Assay kit (Solarbio, Beijing, China). Equal amount of protein was separated in 10% SDS-polyacrylamide gels and electrophoretically transferred onto a polyvinylidene difluoride transfer membrane. After blocking with 5% nonfat milk, the membranes were incubated overnight with specific primary antibodies against β-catenin (Abcam, Shanghai, ab32572), phosphorylated β-catenin (Abcam, Shanghai, ab277785, phosphorylated site S552), and actin (Solarbio, Beijing, China, K200058M). The blots were then visualized by enhanced chemiluminescence (ECL).

**qRT-PCR**: Total RNA (*n* = 5 replicates per group) was extracted with Total RNA Extraction Kit (Solarbio, Shanghai, China), and the quality and concentration were detected using a NanoDrop 2000 spectrophotometer (Thermo Fisher Scientific, Waltham, MA, USA). cDNA was synthesized using the Primescript RT Reagent Kit (TakaRa, Dalian, China). Quantitative real-time PCR (qRT-PCR) analysis was performed using the SYBR Green Master Mix (Roche, Basel, Switzerland). The sequence of primer: WNT6 forward primer GCGGAGACGATGTGGACTTC, reverse primer ATGCACGGATATCTCCACGG; 18S rRNA-CGATGCTCTTAGCTGAGTGT, reverse primer GGTCCAAGAATTTCACCTCT.

**Liquid chromatography–mass spectrometry:** Post treatment (660 nm laser, 10 mW, 1.0 J/cm^2^), the 10 cm dish (*n* = 5 replicates per group, control and treatment) was placed on wet ice, and old culture medium RPMI 1640 was collected to be removed. The cells were washed 3 times with ice-cold NaCl (0.9% in HPLC grade and deionized H_2_O) and frozen on dry ice. Cold 80% MeOH in H_2_O was added to the dish, which was then transferred to wet ice before scrapping the cells. The cells were collected into a clear tube and frozen in liquid nitrogen. Samples were stored at −80 °C until analysis.

Samples were vortexed and left on ice for 10 min. After centrifugation, supernatant was transferred to new tube and evaporated to dryness using speed vacuum. The samples were prepared and analyzed by LC–MS by Novogene company (Tianjin, China). MetaboAnalyst was used to examine significantly changed metabolites and generate heat map, principal coordinate analysis, and volcano plot (www.metaboanalyst.ca/, 1 March 2022).

**Statistical analysis**: Data were analyzed using GraphPad Prism 8.2.1(GraphPad Software, La Jolla, CA, USA) and R language (version 3.6). Volcano plot, heatmap, and bubble plot were generated using Rstudio software. One-way analysis of variance (ANOVA) with Tukey’s test was used for comparison of two groups in validation assay, including qRT-PCR, metabolite analysis, and WB analysis. Both methods were achieved by the “Analyze” function in GraphPad Prism. All data are presented as mean ± SEM (standard error of mean). Statistical significance is considered as follows: * *p* < 0.05, ** *p* < 0.01. If nom *p*-value < 0.05 and NES ≥ 1.0, this means that this pathway can be substantially considered upregulation. If nom *p*-value < 0.05 and NES ≤ −1.0, this means that this pathway can be substantially considered downregulation.

## 3. Results

**Figure 1:**
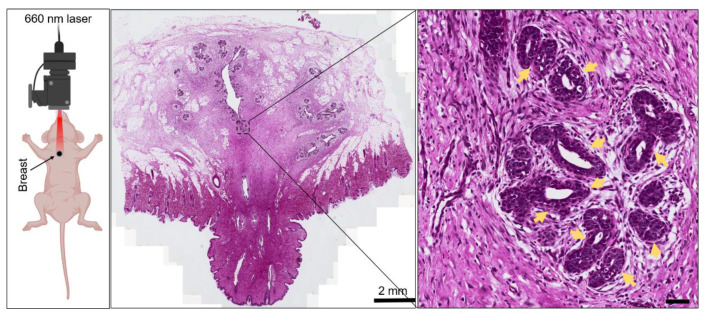
Diagram of mouse breast (*n* = 3 replicates) with exposure to 660 nm laser (power, 10 mW; total light dosage, 10 J/cm^2^). The exposed breast tissue was obtained, and H&E staining was conducted to examine the morphological changes post treatment. Scale bar is 2 mm and 50 μm, respectively.

**Biological impact of breast tissue with long-term exposure to 660 nm**. Murine animals have been commonly employed as therapeutic models to examine therapeutic benefits post intervention, for example, orthotopic human breast cancer implanted in breast tissue [24]. However, most external light or laser interventions always cause abnormalities, for example, retina damage [25] and the thermal effects of lasers on organ development [26]. To determine the biological effects of 660 nm laser exposure, animal- or cell-based assays, including H&E staining, RNA sequencing, bioinformatics analysis, metabolite analysis, and relative molecular biology assays, were conducted. Consequently, we also employed BALB/c mouse to determine the impacts of laser exposure, as given in the left panel of Figure 1.

Here, to examine the biological influences of red light on breast tissues, BALB/c mouse breast with fur removed was exposed to a 660 nm laser (10 mW, 10 J/cm^2^). Post intervention, this breast tissue was resected and further examined using H&E staining. As given in Figure 1, no significant tissue damage was observed by identifying changes in breast tissue morphology. Owing to the highly dense ducts and microvessels in breast tissue, we also examined the integrity of these vascular networks, presented by yellow arrows in the zoomed image. Near the boundary of microvessels, we did not observe any leakage of hemoglobin, which will be presented as high-light red patterns. These results demonstrated that 660 nm laser radiation did not influence the morphology of breast microvessels. Therefore, these results suggest that long-term exposure to a 660 nm laser do not display any significantly histological damage to breast tissue.

**RNA sequencing analysis**: Although H&E staining suggested that no significant damage was observed post intervention, it is not as clear to depict the few impacts of 660 nm laser radiation on breast tissue. Subsequently, we performed 660 nm radiation on mouse epithelial cells, HC11, with two intervention dosages. The HC11 cell line has been widely used to determine the molecular mechanisms of external intervention, for example, 17β-estradiol regulating STAT5 isoforms in female mammary epithelial cells [27] and LGL1 binding to integrin β1 to promote epithelial branching [28]. Owing to the critical roles of HC11 cells in female epithelial cell investigation, we consequently determined a laser intervention on female epithelial cells.

**Figure 2:**
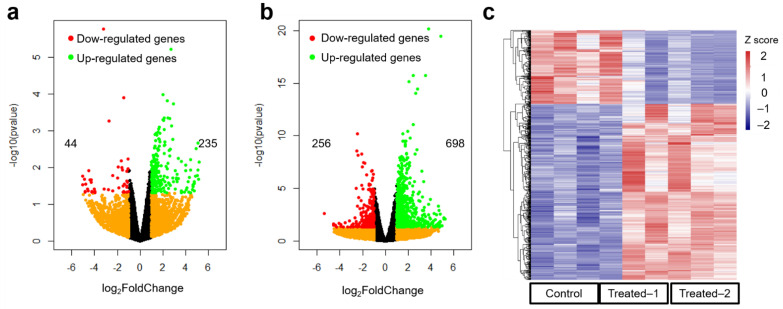
RNA sequencing analysis of HC11 cells post treatment. (**a**). Gene profile of cells (*n* = 3 per group) post treatment. Intervention dosage is set as 0.5 J/cm^2^. In total, 44 genes and 235 genes were identified as downregulated and upregulated differentially expressed genes, respectively. (**b**). Gene profile of cells post treatment. Intervention dosage is set as 1.0 J/cm^2^. In total, 256 genes and 698 genes were identified as downregulated and upregulated differentially expressed genes, respectively. (**c**). Heatmap of differentially expressed genes with different intervention. Gene expression was normalized using z-score method. Threshold value to consider as significant difference was set as FDR q < 0.05 and |log_2_FoldChange| ≥ 1.0.

To precisely examine the gene profiles post long-term exposure to 660 nm on breast vasculature, RNA sequencing experiments were conducted with different intervention dosages, 0.5 J/cm^2^ and 1.0 J/cm^2^, respectively. These intervention doses were set according to Monte Carlo eXtreme simulation, which is presented in Figure S1. Post intervention (Figure 2a,b), there were 279 and 954 genes identified as differentially expressed genes (DEGs), respectively, which may be associated with modulating cellular response to 660 nm laser radiation. For low-dosage intervention (Figure 2a), 44 and 235 genes were identified as downregulated and upregulated DEGs (Table S1), respectively. The amount of upregulated DEGs was ~5-fold compared with downregulated DEGs, implying that upregulated pathways may play a central role in following cellular response. When the intervention dosage was increased to 1.0 J/cm^2^ (Figure 2b), 256 and 698 genes were identified as downregulated and upregulated DEGs (SI Table S2), respectively. Notably, folds of upregulated DEGs were attenuated to ~2.7 compared with downregulated DEGs, demonstrating that upregulated pathways may be highly involved in regulating the biological processes of 660 nm laser radiation for further cellular response. Interestingly, as presented in the heatmap (Figure 2c), we can observe that these DEGs (as given in Table S2) are likely changed depending on the intervention dosage. These results suggest that investigation of RNA sequencing data should be employed to explore which pathways are essential for cellular response to 660 nm laser radiation.

**Figure 3:**
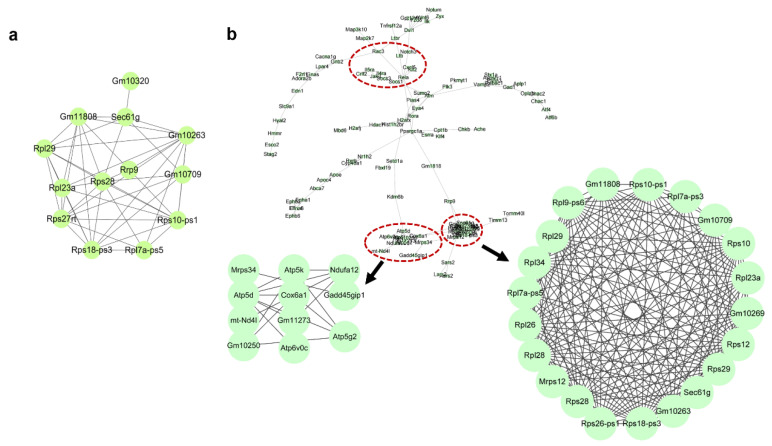
Protein–protein interaction (PPI) network of differentially expressed gene dependence on intervention group: (**a**) low-dosage intervention and (**b**) high-dosage intervention group. The PPI network was produced using STRING and Cytoscape software. Key nodes to modulate relative pathways were labeled using circles with different colors.

**Figure 4:**
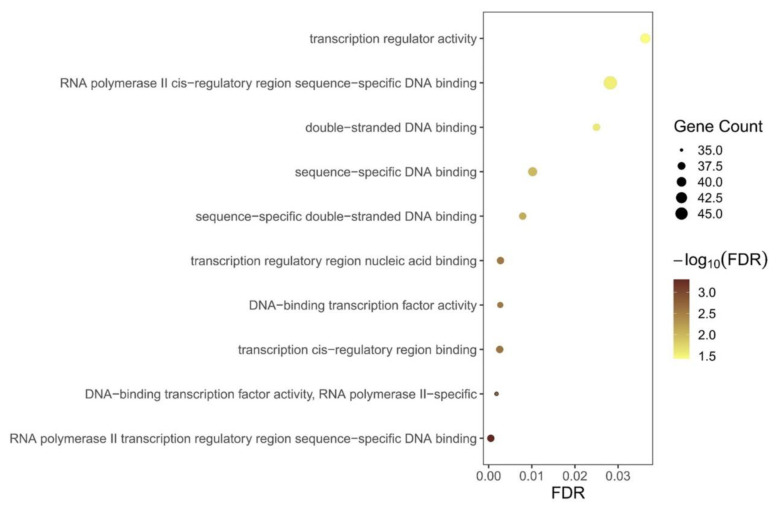
Gene ontology (GO) analysis of differentially expressed genes in the high-dosage intervention group compared with the control group. GO enrichment analysis of differentially expressed genes was retrieved using go:profiler website-based database. All the adjusted statistically significant values of these terms were labeled using negative 10-based log transformed with different colors.

**Protein–protein interaction network and gene ontology analysis:** To further examine how the 660 nm laser intervention affects gene profiles in HC11 cells, upregulated DGEs were employed to conduct protein–protein interaction (PPI) network and gene ontology (GO) analysis to identify critical node genes and pathways. As given in Figure 3a, only 11 genes were identified as a high correlation, and these genes are associated with gene transcription, for example, *Rps28*, *Rps18-ps3*, *Rpl7a-ps5*, *Rps27rt*, *Rps10-ps1*, *Rpl23a*, *Rpl29*, and *Sec61g*. Unfortunately, no hub genes in the low-dosage intervention group were observed compared with the control group, and other bioinformatics analyses failed to present enriched annotations with a statistical difference. When the intervention dosage was increased to 1.0 J/cm^2^, the image of the PPI network analysis (Figure 3b) displayed several clusters labeled with different colored circles, and these clusters can be attributed to transcription, the inflammation response, and the Eph/Ephrin kinase family. Furthermore, we also conducted a GO analysis of DEGs depending on the different intervention groups using the go:profiler database, and only the GO analysis of the high-dosage intervention (Figure 4) is presented as the top 10 terms. These terms can be attributed as transcription regulation-related terms and DNA binding-related terms, consistent with PPI network analysis. Therefore, these results suggest that 660 nm laser intervention mainly affects transcription-related pathways to modulate downstream pathways and display different phenotypes.

**Figure 5:**
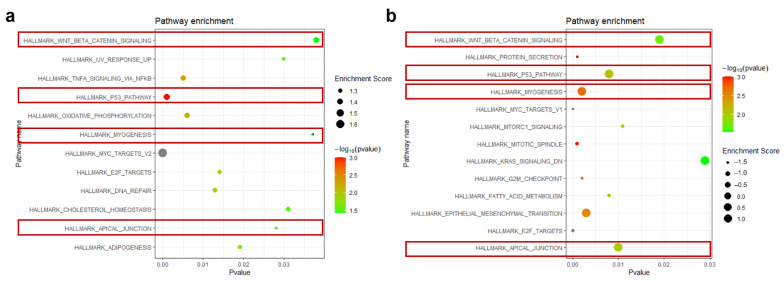
Gene set enrichment analysis (GSEA) depending on different intervention groups: (**a**) low-dosage intervention compared with control group; (**b**). high-dosage intervention compared with control group. GSEA was conducted in GSEA software, and pathways identified in both GSEA are labeled with red squares. Threshold value to consider as significant difference is set as |normalized enrichment score (NES)| ≥ 1.0 and NOM *p*-value < 0.05 and FDR q-value < 0.25.

**Figure 6:**
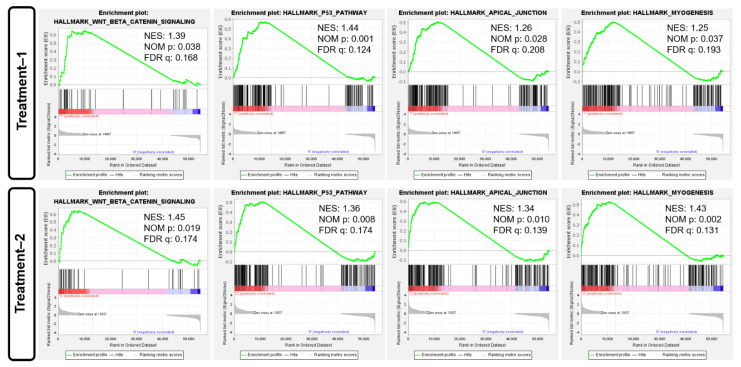
GSEA score profile of four enriched pathways depending on light treatment with two dosages: Hallmark_p53_pathway, Hallmark_Wnt_beta_catenin_signaling, Hallmark_Apical_junction, and Hallmark_Myogenesis. Threshold value to consider as significant difference is set as |normalized enrichment score (NES)| ≥ 1.0 and NOM *p*-value < 0.05 and FDR q-value < 0.25.

**Gene set enrichment analysis (GSEA):** To systematically assess the influence of 660 nm laser intervention, we conducted GSEA depending on different intervention dosages using Hallmark gene sets. As given in Figure 5a, there were 12 enriched pathways considered as having a significant difference, i.e., hallmark_Wnt_beta_catenin_signaling, hallmark_UV_response_up, hallmark_TNFA_signaling_via_NFKB, hallmark_p53_pathway, hallmark_oxidative_phosphorylation, Hallmark_myogenesis, hallmark_myc_targets_v2, hallmark_E2F_targets, hallmark_DNA_repair, hallmark_apical_junction, and hallmark_adipogenesis. Owing that the value of the normalized enrichment score (NES) is more than 1.0, these pathways could be substantially considered upregulation. When the intervention dosage was increased to 1.0 J/cm^2^, 13 enriched pathways (Figure 5b) could be considered as having a significant difference, including 6 substantially upregulated pathways and 7 substantially upregulated pathways. To identify the critical pathways modulating the cellular response, we found that there were four substantially upregulated pathways (Figure 6), which were identified in both enriched terms. These pathways are hallmark_Wnt_beta_catenin_signaling, hallmark_p53_pathway, hallmark_apical_junction, and Hallmark_myogenesis, respectively. As per previous reports, the p53 pathway is also associated with apoptosis [29], and the abovementioned results did not display any apoptotic phenotype, implying that the p53 pathway may not be a critical pathway in modulating the cellular response during 660 nm laser intervention. Moreover, the apical junction [30,31] and myogenesis [32,33] have been corroborated as having a higher association with Wnt_beta_catenin_signaling. Therefore, these results suggest that the Wnt/β-catenin pathway may play a critical role in the cellular response during 660 nm laser intervention.

**Figure 7:**
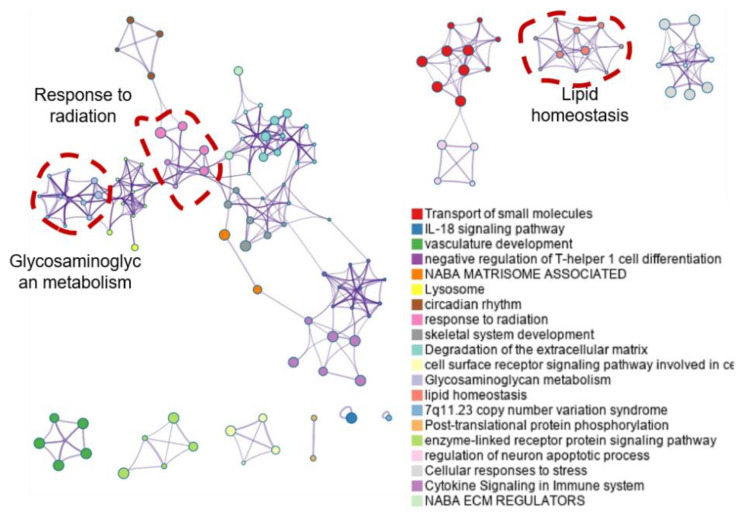
Metascape analysis of enriched pathways using upregulated DEGs in higher intervention dosage.

**External intervention alters metabolites**. As a major external intervention resource, light often involves modulating molecular transduction via affecting Ca^2+^ or other signaling molecules [34,35]. To determine whether light can regulate epithelial cells of breast vessels, we conducted pathway enrichment analysis using Metascape. As given in Figure 7, enriched pathways were clustered as several major nodes, i.e., response to radiation, glycosaminoglycan metabolism, degradation of the extracellular matrix, cytokine signaling in the immune system, transport of small molecules, and lipid homeostasis. Among these enriched pathways, metabolism-related terms, for example, glycosaminoglycan metabolism and lipid homeostasis, were enriched, implying that external light intervention might affect the metabolism of epithelial cells.

**Figure 8:**
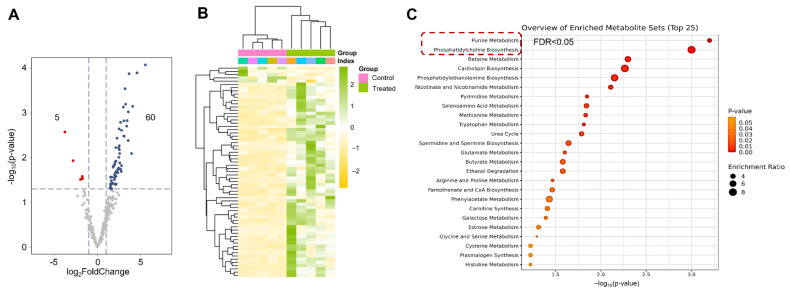
(**A**). Volcano plot of metabolite profiles dependent on log_2_FoldChange (*n* = 5 replicates per group). Red dots are presented as downregulated metabolites, and blue dots are presented as upregulated metabolites (threshold value |log_2_FoldChange| > 1.0 and FDR q-value < 0.05). Intervention dosage is set as 1.0 J/cm^2^. (**B**). Heatmap shows abundance of significant difference in metabolites post intervention compared with the control group. Five parallels per group. Clustering was conducted at row and column levels. (**C**). Metabolism pathways on significantly upregulated metabolites (*p* < 0.05 and FDR q-value < 0.05) in control and treatment groups obtained from MetaboAnalyst website.

Considering the abovementioned bioinformatics analysis, few pathways were enriched post the low intervention (0.5 J/cm^2^), implying that the profile of metabolites under this intervention condition may not be significantly affected. Consequently, metabolism analysis between the higher intervention dosage (1.0 J/cm^2^) and the control was conducted on HC11 cells. Subsequently, metabolites extracted from the HC11 cells post treatment were analyzed using liquid chromatography–mass spectrometry (LC–MS) to examine changes compared with the control group (Table S3). As expected, external light treatment resulted in significant changes to several critical metabolites (Figure 8A). Among these significant changes in metabolites, 5 downregulated and 60 upregulated metabolites were identified, and the relative intensity of these significant changes is represented in a heatmap with group clusters (Figure 8B). Pathway analysis of these significantly changed metabolites (*p*-value < 0.05 and FDR < 0.05) revealed that an increase in enriched pathways is directly associated with purine metabolism and phosphatidylcholine biosynthesis (Figure 8C, labeled by red square). The significantly changed metabolites in enriched phosphatidylcholine biosynthesis are cytidine monophosphate, pyrophosphate (diphosphate), *S*-adenosylmethionine (*S*-adenosyl-L-methionine), and CDP-ethanolamine. These metabolites are mainly located at the endoplasmic reticulum and mitochondria, which catalyze products of nucleotide and lipid synthesis. Moreover, enrichment of purine metabolism displayed a higher association with nucleotide synthesis, consistent with the phosphatidylcholine biosynthesis pathway.

**Figure 9:**
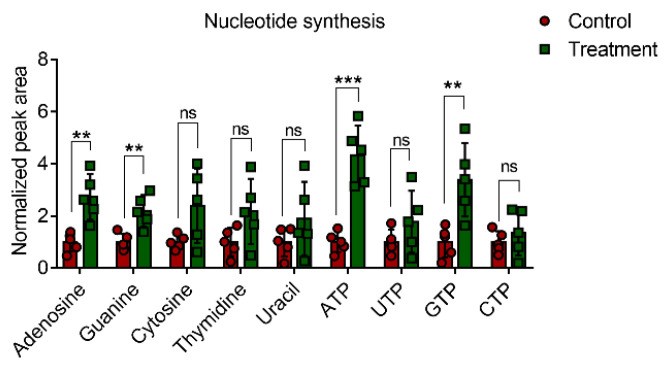
Normalized peak areas of the selected metabolites post treatment. Intervention dosage is set as 1.0 J/cm^2^. Data are presented as mean ± standard error (*n* = 5 per group). One-way analysis of variance (ANOVA) with Tukey’s test: ns, no significance; ** *p* < 0.01; *** *p* < 0.001.

Finally, to further demonstrate how external light intervention affects nucleotide metabolism, we examined several critical nucleotide metabolites post intervention. As given in Figure 9, only adenosine, guanine, ATP, and GTP were significantly upregulated. Except as energy suppliers, adenosine, guanine, and relative metabolites also play an essential role in modulating signaling transduction, for example, the expression of brain-derived neurotrophic factor by cycling adenosine monophosphate [36], critical substrates in the GTPase-related pathway [37,38,39]. These results imply that nucleotide-related second messages might be involved in the response to light-induced stimulus.

**Figure 10:**
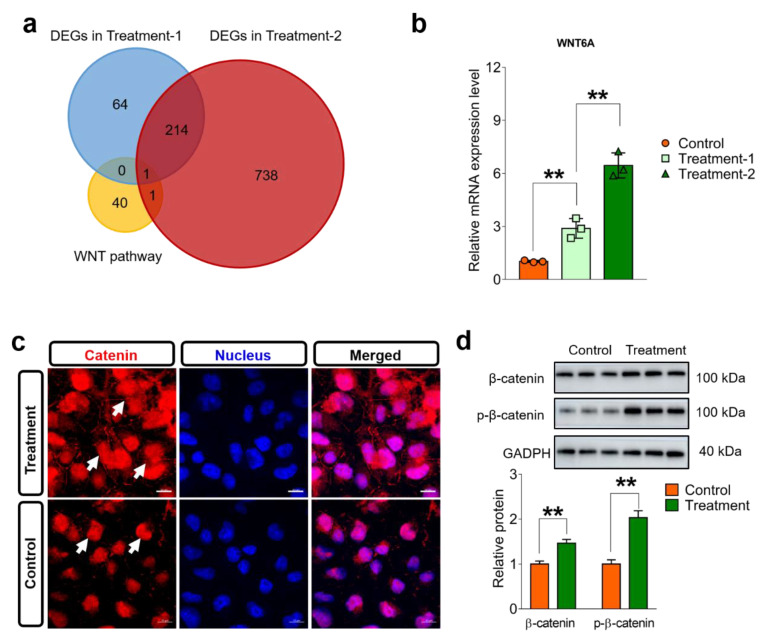
Exposure to 660 nm laser modulates β-catenin-mediated Wnt pathway. (**a**). Venn diagram of Wnt pathway gene set and DEGs depending on different treatment groups. (**b**). Quantification of *WNT6* gene post different intervention. Intervention dosages are set as 0.5 and 1.0 J/cm^2^ (*n* = 3 per group). (**c**). Immunofluorescence staining of catenin protein (red) post treatment (*n* = 3 replicates per group). Nucleus was stained using DAPI dye. Intervention dosage is set as 1.0 J/cm^2^. (**d**). Western blot and quantitative measurement analysis of β-catenin and relative phosphorylation levels post intervention (*n* = 3 replicates per group). Data are presented as mean ± standard error. One-way analysis of variance (ANOVA) with Tukey’s test, **, *p* < 0.01.

**Intervention activates the Wnt/β-catenin pathway**. As mentioned above, the Wnt/β-catenin pathway may be the essential pathway to modulate downstream genes post intervention. Here, we firstly analyzed the overlapped genes among the DEGs in different groups and the gene set of the Wnt/β-catenin pathway, in which the *WNT6* gene was enriched, as given in Figure 10a. Moreover, we also found that 214 genes were identified in both DEGs with different intervention groups (Table S4). To validate the role of the Wnt/β-catenin pathway, qRT-PCR assays were conducted, and the results demonstrated that the *WNT6* gene displayed dosage-dependent upregulation post intervention (Figure 10b). In the Wnt/β-catenin pathway, catenin protein is the key regulatory protein to modulate downstream genes. We firstly examined the distribution of β-catenin in the HC11 cells post intervention, and the confocal images showed (Figure 10c) that β-catenin was significantly upregulated and redistributed to cell membranes, consistent with enrichment of the apical junction pathway. Furthermore, we also conducted Western blot analysis of β-catenin and relative phosphorylation levels. As shown in Figure 10d, we can observe that the high-dosage intervention significantly upregulated expression levels of β-catenin and promoted phosphorylation levels. These results suggest that a high dosage-dependent intervention can activate the Wnt/β-catenin pathway to further modulate transcription levels of downstream genes.

## 4. Discussion

Here, our investigation indicated that breast epithelial cells post 660 nm laser radiation did not significantly affect the cellular morphology, and the RNA sequencing results illustrated that 660 nm laser radiation could activate the Wnt/β-catenin pathway to further modulate the expression levels of downstream genes. These results suggest that long-term exposure to a 660 nm laser may be helpful for breast vessels through the Wnt/β-catenin pathway, requiring more evidence to support this hypothesis.

Radiation therapy with red laser or LED light has been widely used for disease therapy [40,41], especially for photosensitizer-mediated photodynamic interventions [42,43,44]. With low-power and high-dosage intervention, no significant morphological changes were observed, and the integrity of the breast vasculature was not damaged (Figure 1). Although the previous literature has reported that red light could modulate proliferation and apoptosis pathways to induce further cellular response [45,46], no stronger evidence supports that long-term red radiation is harmful to human organisms [47]. No significant morphology damage does not mean that red laser radiation will not further influence the cellular response at molecular levels. Therefore, more evidence of the biological impact post radiation may benefit from RNA sequencing.

By identifying the differentially expressed genes (DEGs) depending on intervention dosage, the number of DEGs was significantly increased from 279 to 954 (Figure 2a,b), implying that dosage-dependent radiation can significantly affect the gene profiles of breast epithelial cells (HC11 cells). Light intervention can generate a thermal effect on cells [48], although intermittent intervention can inhibit this thermal effect. RNA sequencing results revealed that no heatshock-related proteins were significantly upregulated (Figure 2) and illustrated that this type of thermal effect could be inhibited by intermittent intervention. These results are corroborated by GO (Figure 4) and GSEA analyses (Figure 5). Therefore, we should consider other pathways of red laser radiation to modulate the expression levels of downstream genes.

Light, as a common external intervention, often modulates signaling transduction by photoreceptors [49] or by affecting second messages [50]. Owing to the presence of various ligands binding to enzymes, external light can modulate metabolism-related processes, for example, TCA [51] and ATP synthesis [52]. However, our study, by determining the metabolite profiles, revealed that external light could significantly upregulate several metabolites (Figure 8A,B), and enrichment pathway analysis demonstrated that two pathways, i.e., purine metabolism and phosphatidylcholine biosynthesis, were enriched, and bioinformatics analysis based on the roles of these upregulated metabolites confirmed that external light intervention (660 nm) on HC11 cells is nucleotide metabolism. To analyze the profiles of nucleotides using LC–MS, adenosine, guanine, and related triphosphate products were significantly upregulated post intervention (Figure 9). Why did 660 nm light promote products of these metabolites? For ATP and related metabolites, red light can modulate the oxidative phosphorylation pathways [53,54,55], which is a major mechanism for generating ATP. Owing to the salvage pathways of purine synthesis in eukaryotic cells [56], inosine-5′-monophosphate dehydrogenase 1 (*IMPDH1*) may be a critical node for modulating guanine nucleotides by external illumination conditions. However, several nucleotide moieties (cAMP, GTP, GDP, or GMP) are important second messages for preserving signaling transduction. The concentration of these nucleotide moieties in vivo may involve regulating downstream pathways, which requires more investigation to identify the impact of a 660 nm laser on signaling transduction.

Owing to the presence of cytochrome-related proteins, the previous literature has proven that light can modulate the biological functions of mitochondria and further regulate downstream pathways [57,58,59]. Bioinformatics analysis revealed that four pathways, i.e., hallmark_Wnt_beta_catenin_signaling, hallmark_p53_pathway, hallmark_apical_junction, and Hallmark_myogenesis, were observed in both GSEA analysis results. Considering the high association between mitochondria and the Wnt/β-catenin pathway [60,61], we can believe that 660 nm laser radiation can substantially activate the Wnt/β-catenin pathway. We conducted RT-PCR, immunofluorescence staining, and Western blot analysis, and the results strongly suggested that 660 nm laser radiation activates the Wnt/β-catenin pathway through upregulating the WNT6 gene, β-catenin, and relative phosphorylation levels.

The Wnt/β-catenin pathway is involved in many biological processes, including modulating chronic inflammation [62], liver functions [63], regulating cell proliferation [64], genome stability [65], neurodegeneration [66], and cardiometabolic disorders [67]. Upregulating the Wnt/β-catenin pathway may be of great impact on breast vessel functions, including uremic vascular calcifications [68] and angiogenesis [69,70,71]. However, most vessel damage also induces downregulation of the Wnt/β-catenin pathway, and complementary Wnt sources can promote vascular development post damage [72]. Activation of the Wnt/β-catenin pathway led by a 660 nm laser may provide potential intervention for vascular repair.

## 5. Conclusions

In summary, H&E staining of breast tissue post intervention did not display any significant damage and microvessel leakage. Through omics analysis by RNA sequencing and relative bioinformatics, we found that 660 nm laser intervention can significantly affect the expression levels of transcription-related genes, and further GO functional analysis revealed that the Wnt/β-catenin pathway can be considered substantial upregulation. These results were corroborated by qRT-PCR, immunofluorescence staining, and Western blot analysis. Significant changes in metabolites and pathway analysis revealed that a 660 nm laser could affect nucleotide metabolism by regulating purine metabolism. Our findings suggest that 660 nm laser radiation may activate the Wnt/β-catenin pathway and nucleotide metabolism, which provides a potential approach to identifying the impact of a 660 nm laser on the microcirculation system.

## Data Availability

Data are available on request from the authors.

## Supplementary Materials

The following supporting information can be downloaded at: https://www.mdpi.com/article/10.3390/biom12101389/s1, Figure S1: Photon migration within breast tissues. Scale bar is energy density (J/cm^2^). Red raw is the light direction and grey square is light source.; Table S1: Downregulated and upregulated DEGs in first group; Table S2: Downregulated and upregulated DEGs in second group; Table S3: Metabolite profiles in control and treated groups; Table S4: 214 genes were identified in both DEGs with different intervention groups [73,74,75,76].

## Abbreviations

**qRT-PCR**	Real-time quantitative reverse transcription PCR
**GO**	Gene ontology
**PPI**	Protein–protein interaction
**GSEA**	Gene set enrichment analysis
**DEGs**	Differentially expressed genes
**DAPI**	4′,6′-diamidino-2-phenylindole
**LED**	Light-emitting diode
**NES**	Normalized enrichment score
**LC–MS**	Liquid chromatography–mass spectrometry
**PPI**	Protein–protein interaction

## References

[1] Wajih N., Alipour E., Rigal F., Zhu J., Perlegas A., Caudell D.L., Kim-Shapiro D. (2021). Effects of nitrite and far-red light on coagulation. Nitric Oxide Biol. Chem..

[2] Wang L., Ma R., Liu C., Liu H., Zhu R., Guo S., Tang M., Li Y., Niu J., Fu M. (2017). Salvia miltiorrhiza: A Potential Red Light to the Development of Cardiovascular Diseases. Curr. Pharm. Des..

[3] Wu Y., Deng Y., Huang P. (2021). Application of red light therapy for moderate-to-severe acne vulgaris: A systematic review and meta-analysis. J. Cosmet. Dermatol..

[4] Kwiatkowski S., Knap B., Przystupski D., Saczko J., Kędzierska E., Knap-Czop K., Kotlińska J., Michel O., Kotowski K., Kulbacka J. (2018). Photodynamic therapy—Mechanisms, photosensitizers and combinations. Biomed. Pharmacother. Biomed. Pharmacother..

[5] Núñez-Álvarez C., Del Olmo-Aguado S., Merayo-Lloves J., Osborne N.N. (2017). Near infra-red light attenuates corneal endothelial cell dysfunction in situ and in vitro. Exp. Eye Res..

[6] Richardson C., Cain N., Bartel K., Micic G., Maddock B., Gradisar M. (2018). A randomised controlled trial of bright light therapy and morning activity for adolescents and young adults with Delayed Sleep-Wake Phase Disorder. Sleep Med..

[7] Bashkatov A.N., Berezin K.V., Dvoretskiy K.N., Chernavina M.L., Genina E.A., Genin V.D., Kochubey V.I., Lazareva E.N., Pravdin A.B., Shvachkina M.E. (2018). Measurement of tissue optical properties in the context of tissue optical clearing. J. Biomed. Opt..

[8] El Nawam H., El Backly R., Zaky A., Abdallah A. (2019). Low-level laser therapy affects dentinogenesis and angiogenesis of in vitro 3D cultures of dentin-pulp complex. Lasers Med. Sci..

[9] das Neves L.M., Leite G.P., Marcolino A.M., Pinfildi C.E., Garcia S.B., de Araújo J.E., Guirro E.C. (2017). Laser photobiomodulation (830 and 660 nm) in mast cells, VEGF, FGF, and CD34 of the musculocutaneous flap in rats submitted to nicotine. Lasers Med. Sci..

[10] Wang Z.X., Kim S.H. (2020). Effect of Photobiomodulation Therapy (660 nm) on Wound Healing of Rat Skin Infected by Staphylococcus. Photobiomodulation Photomed. Laser Surg..

[11] Ebrahimpour-Malekshah R., Amini A., Zare F., Mostafavinia A., Davoody S., Deravi N., Rahmanian M., Hashemi S.M., Habibi M., Ghoreishi S.K. (2020). Combined therapy of photobiomodulation and adipose-derived stem cells synergistically improve healing in an ischemic, infected and delayed healing wound model in rats with type 1 diabetes mellitus. BMJ Open Diabetes Res. Care.

[12] Liu J., Chen C., Wei T., Gayet O., Loncle C., Borge L., Dusetti N., Ma X., Marson D., Laurini E. (2021). Dendrimeric Nanosystem Consistently Circumvents Heterogeneous Drug Response and Resistance in Pancreatic Cancer. Exploration.

[13] Bostanciklioglu M., Demiryürek Ş., Cengiz B., Demir T., Öztuzcu S., Aras M.H., Özsevik S., Usumez A., Ergün S., Özbal H.K. (2015). Assessment of the effect of laser irradiations at different wavelengths (660, 810, 980, and 1064 nm) on autophagy in a rat model of mucositis. Lasers Med. Sci..

[14] de Medeiros M.L., Araújo-Filho I., da Silva E.M., de Sousa Queiroz W.S., Soares C.D., de Carvalho M.G., Maciel M.A. (2017). Effect of low-level laser therapy on angiogenesis and matrix metalloproteinase-2 immunoexpression in wound repair. Lasers Med. Sci..

[15] Rajendran N.K., Houreld N.N., Abrahamse H. (2021). Photobiomodulation reduces oxidative stress in diabetic wounded fibroblast cells by inhibiting the FOXO1 signaling pathway. J. Cell Commun. Signal..

[16] Lee J.H., Chiang M.H., Chen P.H., Ho M.L., Lee H.E., Wang Y.H. (2018). Anti-inflammatory effects of low-level laser therapy on human periodontal ligament cells: In vitro study. Lasers Med. Sci..

[17] Kaviani A., Fateh M., Ataie-Fashtami L., Yunesian M., Najafi M., Berry M., Rabbani A. (2008). Comparison of carbon dioxide laser and scalpel for breast lumpectomy: A randomized controlled trial. Photomed. Laser Surg..

[18] Trelles M.A., Pardo L., Chamorro J.J., Bonanad E., Allones I., Buil C., Luna R. (2005). Erbium: YAG laser as a method of deepithelization in corrective and reductive breast surgery. Ann. Plast. Surg..

[19] Ahcan U., Bracun D., Zivec K., Pavlic R., Butala P. (2012). The use of 3D laser imaging and a new breast replica cast as a method to optimize autologous breast reconstruction after mastectomy. Breast.

[20] Miller K.D., Nogueira L., Devasia T., Mariotto A.B., Yabroff K.R., Jemal A., Kramer J., Siegel R.L. (2022). Cancer treatment and survivorship statistics, 2022. CA A Cancer J. Clin..

[21] Katzenellenbogen J.A., Johnson H.J., Carlson K.E., Myers H.N. (1974). Photoreactivity of some light-sensitive estrogen derivatives. Use of an exchange assay to determine their photointeraction with the rat uterine estrogen binding protein. Biochemistry.

[22] Liu C., Miyajima T., Melangath G., Miyai T., Vasanth S., Deshpande N., Kumar V., Ong Tone S., Gupta R., Zhu S. (2020). Ultraviolet A light induces DNA damage and estrogen-DNA adducts in Fuchs endothelial corneal dystrophy causing females to be more affected. Proc. Natl. Acad. Sci. USA.

[23] Anders S., Huber W. (2010). Differential expression analysis for sequence count data. Genome Biol..

[24] Rashid O.M., Nagahashi M., Ramachandran S., Dumur C., Schaum J., Yamada A., Terracina K.P., Milstien S., Spiegel S., Takabe K. (2014). An improved syngeneic orthotopic murine model of human breast cancer progression. Breast Cancer Res. Treat..

[25] Pollack J.S., Kim J.E., Pulido J.S., Burke J.M. (1998). Tissue effects of subclinical diode laser treatment of the retina. Arch. Ophthalmol..

[26] Chuck R.S., Behrens A., Wellik S., Liaw L.L., Dolorico A.M., Sweet P., Chao L.C., Osann K.E., McDonnell P.J., Berns M.W. (2001). Re-epithelialization in cornea organ culture after chemical burns and excimer laser treatment. Arch. Ophthalmol..

[27] Jallow F., Brockman J.L., Helzer K.T., Rugowski D.E., Goffin V., Alarid E.T., Schuler L.A. (2018). 17 β-Estradiol and ICI182, 780 Differentially Regulate STAT5 Isoforms in Female Mammary Epithelium, With Distinct Outcomes. J. Endocr. Soc..

[28] Ma R., Gong D., You H., Xu C., Lu Y., Bergers G., Werb Z., Klein O.D., Petritsch C.K., Lu P. (2022). LGL1 binds to Integrin β1 and inhibits downstream signaling to promote epithelial branching in the mammary gland. Cell Rep..

[29] Oren M. (2003). Decision making by p53: Life, death and cancer. Cell Death Differ..

[30] Chen B., Lan J., Xiao Y., Liu P., Guo D., Gu Y., Song Y., Zhong Q., Ma D., Lei P. (2021). Long noncoding RNA TP53TG1 suppresses the growth and metastasis of hepatocellular carcinoma by regulating the PRDX4/β-catenin pathway. Cancer Lett..

[31] Bautista-García P., Reyes J.L., Martín D., Namorado M.C., Chavez-Munguía B., Soria-Castro E., Huber O., González-Mariscal L. (2013). Zona occludens-2 protects against podocyte dysfunction induced by ADR in mice. Am. J. Physiol.-Ren. Physiol..

[32] Kazanskaya O., Glinka A., del Barco Barrantes I., Stannek P., Niehrs C., Wu W. (2004). R-Spondin2 is a secreted activator of Wnt/β-catenin signaling and is required for Xenopus myogenesis. Dev. Cell.

[33] Nakamura T., Sano M., Songyang Z., Schneider M.D. (2003). A Wnt-and β-catenin-dependent pathway for mammalian cardiac myogenesis. Proc. Natl. Acad. Sci. USA.

[34] Hardie R.C., Minke B. (1992). The trp gene is essential for a light-activated Ca^2+^ channel in Drosophila photoreceptors. Neuron.

[35] Golovynska I., Golovynskyi S., Stepanov Y.V., Stepanova L.I., Qu J., Ohulchanskyy T.Y. (2021). Red and near-infrared light evokes Ca^2+^ influx, endoplasmic reticulum release and membrane depolarization in neurons and cancer cells. J. Photochem. Photobiol. B Biol..

[36] Wang Y., Kou S., Gu L., Zheng Q., Li M., Qi F., Zhao H., Wang L. (2012). Effects of Zuogui Pill () and Yougui Pill () on the expression of brain-derived neurotrophic factor and cyclic adenosine monophosphate/protein kinase A signaling transduction pathways of axonal regeneration in model rats with experimental autoimmune encephalomyelitis. Chin. J. Integr. Med..

[37] Gong B., Shen W., Xiao W., Meng Y., Meng A., Jia S. (2017). The Sec14-like phosphatidylinositol transfer proteins Sec14l3/SEC14L2 act as GTPase proteins to mediate Wnt/Ca^2+^ signaling. eLife.

[38] Sinha S., Yang W. (2008). Cellular signaling for activation of Rho GTPase Cdc42. Cell. Signal..

[39] Agarwal R., Jurisica I., Mills G.B., Cheng K.W. (2009). The emerging role of the RAB25 small GTPase in cancer. Traffic.

[40] Jiang C., Li H.T., Zhou Y.M., Wang X., Wang L., Liu Z.Q. (2018). Cardiac optogenetics: A novel approach to cardiovascular disease therapy. Ep Eur..

[41] Mongardini C., Di Tanna G.L., Pilloni A. (2014). Light-activated disinfection using a light-emitting diode lamp in the red spectrum: Clinical and microbiological short-term findings on periodontitis patients in maintenance. A randomized controlled split-mouth clinical trial. Lasers Med. Sci..

[42] Grant W., MacRobert A., Bown S., Hopper C., Speight P. (1993). Photodynamic therapy of oral cancer: Photosensitisation with systemic aminolaevulinic acid. Lancet.

[43] Banci H., Strazzi-Sahyon H., Duarte M., Cintra L., Gomes-Filho J., Chalub L., Berton S., de Oliveira V., Dos Santos P., Sivieri-Araujo G. (2020). Influence of photodynamic therapy on bond strength and adhesive interface morphology of MTA based root canal sealer to different thirds of intraradicular dentin. Photodiagnosis Photodyn. Ther..

[44] Ziqi W., Kai C., Costabel U., Xiaoju Z. (2022). Nanotechnology-Facilitated Vaccine Development During the Coronavirus Disease 2019 (COVID-19) Pandemic. Exploration.

[45] Niu T., Tian Y., Cai Q., Ren Q., Wei L. (2015). Red light combined with blue light irradiation regulates proliferation and apoptosis in skin keratinocytes in combination with low concentrations of curcumin. PLoS ONE.

[46] Niu T., Tian Y., Wang G., Guo G., Tong Y., Shi Y. (2020). Inhibition of ROS-NF-κB-dependent autophagy enhances Hypocrellin A united LED red light-induced apoptosis in squamous carcinoma A431 cells. Cell. Signal..

[47] Song S., Zhang Y., Fong C.-C., Tsang C.-H., Yang Z., Yang M. (2003). cDNA microarray analysis of gene expression profiles in human fibroblast cells irradiated with red light. J. Investig. Dermatol..

[48] Zhang J., Yue X., Luo H., Jiang W., Mei Y., Ai L., Gao G., Wu Y., Yang H., An J. (2019). Illumination with 630 nm red light reduces oxidative stress and restores memory by photo-activating catalase and formaldehyde dehydrogenase in SAMP8 mice. Antioxid. Redox Signal..

[49] Ma C., Dai S. (2019). Advances in photoreceptor-mediated signaling transduction in flowering time regulation. Chin. Bull. Bot..

[50] Jakobs K.H., Gierschik P., Grandt R., Marquetant R., Strasser R.H. (1987). Signal Transduction by the Adenylate Cyclase System. Signal Transduction and Protein Phosphorylation.

[51] Gemel J., Randall D.D. (1992). Light regulation of leaf mitochondrial pyruvate dehydrogenase complex: Role of photorespiratory carbon metabolism. Plant Physiol..

[52] Ferraresi C., Kaippert B., Avci P., Huang Y.Y., de Sousa M.V., Bagnato V.S., Parizotto N.A., Hamblin M.R. (2015). Low-level laser (light) therapy increases mitochondrial membrane potential and ATP synthesis in C2C12 myotubes with a peak response at 3–6 h. Photochem. Photobiol..

[53] Yamauchi N., Minagawa E., Imai K., Kobuchi K., Li R., Taguchi Y., Umeda M. (2022). High-Intensity Red Light-Emitting Diode Irradiation Suppresses the Inflammatory Response of Human Periodontal Ligament Stem Cells by Promoting Intracellular ATP Synthesis. Life.

[54] Pastore D.D., Martino C.D., Bosco G., Passarella S. (1996). Stimulation of ATP synthesis via oxidative phosphorylation in wheat mitochondria irradiated with helium-neon laser. IUBMB Life.

[55] Guo S., Li K., Hu B., Li C., Zhang M., Hussain A., Wang X., Cheng Q., Yang F., Ge K. (2021). Membrane-Destabilizing Ionizable Lipid Empowered Imaging—Guided Sirna Delivery and Cancer Treatment. Exploration.

[56] Plana-Bonamaisó A., López-Begines S., Fernández-Justel D., Junza A., Soler-Tapia A., Andilla J., Loza-Alvarez P., Rosa J.L., Miralles E., Casals I. (2020). Post-translational regulation of retinal IMPDH1 in vivo to adjust GTP synthesis to illumination conditions. eLife.

[57] Núñez-Álvarez C., Suárez-Barrio C., Del Olmo Aguado S., Osborne N.N. (2019). Blue light negatively affects the survival of ARPE19 cells through an action on their mitochondria and blunted by red light. Acta Ophthalmol..

[58] Osborne N.N., Núñez-Álvarez C., Del Olmo-Aguado S., Merrayo-Lloves J. (2017). Visual light effects on mitochondria: The potential implications in relation to glaucoma. Mitochondrion.

[59] del Olmo-Aguado S., Manso A.G., Osborne N.N. (2012). Light might directly affect retinal ganglion cell mitochondria to potentially influence function. Photochem. Photobiol..

[60] Ming M., Wang S., Wu W., Senyuk V., Le Beau M.M., Nucifora G., Qian Z. (2012). Activation of Wnt/β-catenin protein signaling induces mitochondria-mediated apoptosis in hematopoietic progenitor cells. J. Biol. Chem..

[61] Shares B.H., Busch M., White N., Shum L., Eliseev R.A. (2018). Active mitochondria support osteogenic differentiation by stimulating β-catenin acetylation. J. Biol. Chem..

[62] Vallée A., Lecarpentier Y. (2018). Crosstalk Between Peroxisome Proliferator-Activated Receptor Gamma and the Canonical WNT/β-Catenin Pathway in Chronic Inflammation and Oxidative Stress During Carcinogenesis. Front. Immunol..

[63] Perugorria M.J., Olaizola P., Labiano I., Esparza-Baquer A., Marzioni M., Marin J.J.G., Bujanda L., Banales J.M. (2019). Wnt-β-catenin signalling in liver development, health and disease. Nat. Rev. Gastroenterol. Hepatol..

[64] Alonso L., Fuchs E. (2003). Stem cells in the skin: Waste not, Wnt not. Genes Dev..

[65] Alberici P., Fodde R. (2006). The role of the APC tumor suppressor in chromosomal instability. Genome Dyn..

[66] Okerlund N.D., Cheyette B.N. (2011). Synaptic Wnt signaling-a contributor to major psychiatric disorders?. J. Neurodev. Disord..

[67] Schinner S. (2009). Wnt-signalling and the metabolic syndrome. Horm. Metab. Res. Horm. Und Stoffwechs. Horm. Et Metab..

[68] Yang C.Y., Chang Z.F., Chau Y.P., Chen A., Yang W.C., Yang A.H., Lee O.K. (2015). Circulating Wnt/β-catenin signalling inhibitors and uraemic vascular calcifications. Nephrol. Dial. Transplant. Off. Publ. Eur. Dial. Transpl. Assoc. Eur. Ren. Assoc..

[69] Benz F., Wichitnaowarat V., Lehmann M., Germano R.F., Mihova D., Macas J., Adams R.H., Taketo M.M., Plate K.H., Guérit S. (2019). Low wnt/β-catenin signaling determines leaky vessels in the subfornical organ and affects water homeostasis in mice. eLife.

[70] Wang L.P., Pan J., Li Y., Geng J., Liu C., Zhang L.Y., Zhou P., Tang Y.H., Wang Y., Zhang Z. (2021). Oligodendrocyte precursor cell transplantation promotes angiogenesis and remyelination via Wnt/β-catenin pathway in a mouse model of middle cerebral artery occlusion. J. Cereb. Blood Flow Metab. Off. J. Int. Soc. Cereb. Blood Flow Metab..

[71] Qiao N., Du G., Zhong X., Sun X. (2021). Recombinant Lactic Acid Bacteria as Promising Vectors for Mucosal Vaccination. Exploration.

[72] Cha B., Geng X., Mahamud M.R., Zhang J.Y., Chen L., Kim W., Jho E.H., Kim Y., Choi D., Dixon J.B. (2018). Complementary Wnt Sources Regulate Lymphatic Vascular Development via PROX1-Dependent Wnt/β-Catenin Signaling. Cell Rep..

[73] Fang Q., Kaeli D.R. (2012). Accelerating mesh-based Monte Carlo method on modern CPU architectures. Biomed. Opt. Express.

[74] Dogdas B., Stout D., Chatziioannou A.F., Leahy R.M. (2007). Digimouse: A 3D whole body mouse atlas from CT and cryosection data. Phys. Med. Biol..

[75] Cheong W.-F., Prahl S.A., Welch A.J. (1990). A review of the optical properties of biological tissues. IEEE J. Quantum Electron..

[76] Strangman G., Franceschini M.A., Boas D.A. (2003). Factors affecting the accuracy of near-infrared spectroscopy concentration calculations for focal changes in oxygenation parameters. Neuroimage.

