# Supplementary material for: Activation of Wnt/β-Catenin Signaling Involves 660 nm Laser Radiation on Epithelium and Modulates Lipid Metabolism
Source: Biomolecules. 2022 Sep 29;12(10):1389. doi: 10.3390/biom12101389 (PMC9599573; doi:10.3390/biom12101389)
Supplement: Supplementary file 1 [file biomolecules-12-01389-s001.zip › biomolecules-1880861-supplementary.pdf]

Photon transport simulation: To determine the migration of photon scattering within mouse breast or skin tissues, Monte Carlo eXtreme (MCX)[1], software running onto Matlab2019 table with GPU acceleration, was employed using digital mouse body data[2]. All the photonic parameters[3-4] were set according to previous report, except especially mentioned. We defined the number of photons as 1.60×10^{8}, and simulation time-step is 5×10^{-6} s. Source type of light is set as planar, and area of light is set as 20×20 unit. All the images were generated using MATLAB software. Post simulation, results showed that 1.64×10^{7} photons were adsorbed by mouse body, indicating that adsorptive coefficacy of 660 nm laser within tissue is ~10%. Consequently, intervention doses applied HC11 cells are set as 0.5 and 1.0 J/cm^2, respectively.

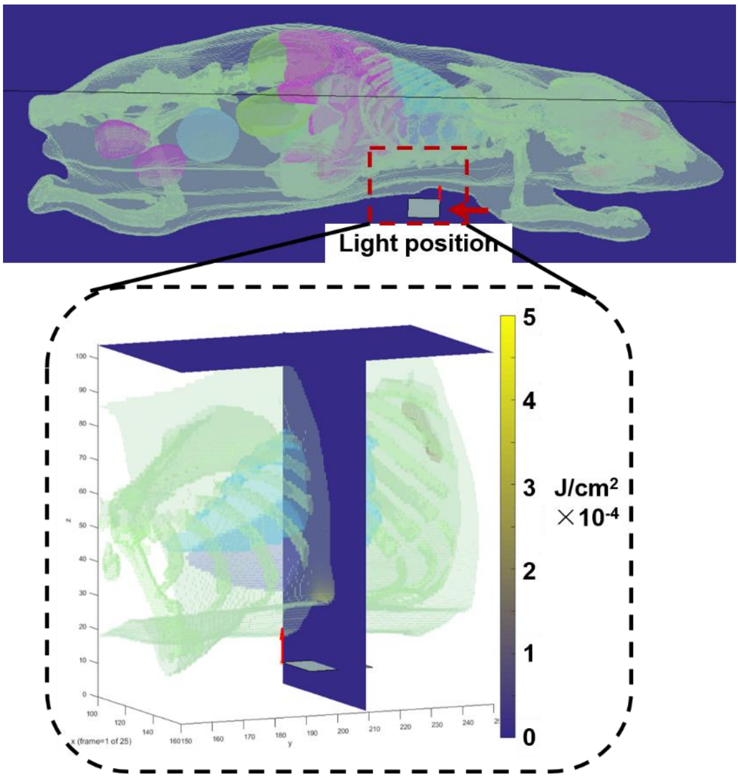

SI Fig.S1. Photon migration within breast tissues. Scale bar is energy density (J/cm^2). Red raw is the light direction and grey square is light source.

References

1. Fang, Q.; Kaeli, D. R., Accelerating mesh-based Monte Carlo method on modern CPU architectures. *Biomedical optics express* **2012,** *3* (12), 3223-3230.

2. Dogdas, B.; Stout, D.; Chatziioannou, A. F.; Leahy, R. M., Digimouse: a 3D whole body mouse atlas from CT and cryosection data. *Physics in Medicine & Biology* **2007,** *52* (3), 577.

3. Cheong, W.-F.; Prahl, S. A.; Welch, A. J., A review of the optical properties of biological tissues. *IEEE journal of quantum electronics* **1990,** *26* (12), 2166-2185.

4. Strangman, G.; Franceschini, M. A.; Boas, D. A., Factors affecting the accuracy of near-infrared spectroscopy concentration calculations for focal changes in oxygenation parameters. *Neuroimage* **2003,** *18* (4), 865-879.

Table S1. Downregulated and upregulated DEGs in first group.

gene	log2FoldChange	pvalue	padj
Gm7769	-5.038	0.030022	0.999608
Gm31191	-5.02544	0.017019	0.999608
Gm43915	-4.85817	0.020725	0.999608
Gm42729	-4.81631	0.046078	0.999608
Oprl1	-4.80575	0.045019	0.999608
Gm37105	-4.79614	0.028746	0.999608
Cyp4a32	-4.62855	0.039428	0.999608
Gm46328	-4.56136	0.043962	0.999608
n-R5s67	-4.51762	0.012143	0.999608
Gm37370	-4.3851	0.022046	0.999608
Gm43920	-4.30797	0.025438	0.999608
Gm13690	-4.30163	0.021556	0.999608
Kncn	-4.24426	0.045621	0.999608
Gm44251	-4.2334	0.046533	0.999608
Mir684-1	-4.03401	0.038602	0.999608
Gm43484	-3.96798	0.045715	0.999608
Gm38397	-3.94126	0.048343	0.999608
Got2-ps1	-3.20511	1.74E-06	0.042947
Gm23468	-3.14582	0.038778	0.999608
Ifit1	-2.76736	0.039845	0.999608
Gm11007	-2.73678	0.000547	0.999608
Ecm2	-2.41492	0.043711	0.999608
Gm18537	-2.39021	0.016463	0.999608
Spef2	-1.97768	0.019057	0.999608
AC122821.1	-1.91467	0.009845	0.999608
Gm9517	-1.84114	0.035561	0.999608
Gm2541	-1.6594	0.006711	0.999608
Zfp85	-1.58285	0.009979	0.999608
Zc3h6	-1.56608	0.011978	0.999608
Gm527	-1.4771	0.046762	0.999608
2210418O10Rik	-1.4699	0.031691	0.999608
Gm19510	-1.4433	0.044925	0.999608
Rps27rt	-1.42865	0.000128	0.775193
9330162G02Rik	-1.40443	0.046266	0.999608
Mt2	-1.38946	0.028211	0.999608
Gm14269	-1.18822	0.023094	0.999608
Gm14401	-1.18023	0.047811	0.999608
Sec24a	-1.15291	0.032045	0.999608
Gm6712	-1.1431	0.012974	0.999608
Gm43857	-1.14138	0.034724	0.999608
Gm48990	-1.1124	0.025069	0.999608

Sec23a	-1.06567	0.010749	0.999608
5430403G16Rik	-1.04917	0.005951	0.999608
Gm13136	-1.01744	0.045246	0.999608
Tarbp2	1.000943	0.007166	0.999608
Rce1	1.003846	0.008792	0.999608
Gm11273	1.009885	0.020619	0.999608
Emc10	1.012964	0.016817	0.999608
Snrnp35	1.019319	0.021826	0.999608
Zfp703	1.027736	0.029975	0.999608
Tlnrd1	1.031897	0.014926	0.999608
Ino80b	1.035713	0.039206	0.999608
Spns1	1.038064	0.023113	0.999608
Fbxl19	1.043656	0.035203	0.999608
Gm11808	1.045766	0.00895	0.999608
1-Sep	1.048501	0.047535	0.999608
Gm14681	1.049137	0.011572	0.999608
Foxp4	1.054752	0.011051	0.999608
Tspan4	1.057164	0.010908	0.999608
Plpp2	1.060442	0.012454	0.999608
Ogfod2	1.060722	0.048883	0.999608
Srf	1.062205	0.027532	0.999608
Klf16	1.062241	0.016582	0.999608
Rps28	1.062757	0.01669	0.999608
Hmga1b	1.065337	0.018084	0.999608
Dusp8	1.066936	0.034624	0.999608
Cldn3	1.068976	0.037154	0.999608
Sox4	1.069002	0.022383	0.999608
Zbtb12	1.072739	0.031449	0.999608
Tmem80	1.073239	0.013296	0.999608
Rabac1	1.081467	0.014866	0.999608
Hnrnpa0	1.086063	0.012994	0.999608
Rai1	1.087237	0.015378	0.999608
Wnt6	1.089431	0.036406	0.999608
Armc5	1.096876	0.006534	0.999608
Ttyh3	1.098746	0.016745	0.999608
Gm10443	1.100531	0.007082	0.999608
6330403L08Rik	1.100644	0.019046	0.999608
Cdh24	1.100766	0.040909	0.999608
Mien1	1.110464	0.046519	0.999608
H1fx	1.111513	0.029034	0.999608
Esrra	1.113811	0.018236	0.999608
Epn1	1.121112	0.016657	0.999608
Sphk1	1.12345	0.040725	0.999608

Gm10282	1.124356	0.038481	0.999608
Dus3l	1.127563	0.028503	0.999608
Atf5	1.136741	0.02118	0.999608
Gm11703	1.137008	0.011164	0.999608
Slc35e4	1.137857	0.020421	0.999608
Gm3788	1.140142	0.026762	0.999608
Ddit4	1.140459	0.02877	0.999608
Rrp9	1.140597	0.016878	0.999608
Atp6v0c	1.14357	0.045265	0.999608
Lingo1	1.151996	0.032057	0.999608
Fam219a	1.156886	0.026011	0.999608
Atf6b	1.157122	0.004662	0.999608
Adamtsl5	1.163976	0.021552	0.999608
1810032O08Rik	1.166527	0.017018	0.999608
Clcf1	1.174713	0.019195	0.999608
Rpp25l	1.175186	0.04947	0.999608
Tmem259	1.180454	0.004844	0.999608
Tomm40l	1.18298	0.025499	0.999608
Capn15	1.183215	0.006846	0.999608
Nradd	1.185965	0.01479	0.999608
Psd	1.190934	0.029446	0.999608
Tspo	1.195764	0.013976	0.999608
Map3k10	1.197099	0.010057	0.999608
Zmynd15	1.206119	0.03942	0.999608
Iba57	1.210154	0.009689	0.999608
Lemd2	1.210838	0.026853	0.999608
Maz	1.211049	0.022996	0.999608
H2afx	1.211824	0.012199	0.999608
Gm12960	1.211938	0.005403	0.999608
Mfsd10	1.213438	0.02222	0.999608
Gm12411	1.213628	0.026691	0.999608
BC037034	1.214809	0.014096	0.999608
Kcnc3	1.219128	0.009774	0.999608
Nr2f6	1.233392	0.011629	0.999608
Rps10-ps1	1.23776	0.018985	0.999608
Ppp2r3d	1.241025	0.009432	0.999608
Ssbp4	1.248579	0.004695	0.999608
Gm15682	1.253588	0.02562	0.999608
Gm8129	1.255643	0.00291	0.999608
Zfp771	1.278516	0.009269	0.999608
Oplah	1.289056	0.014418	0.999608
Ptms	1.292454	0.006217	0.999608
Gm6485	1.299634	0.028325	0.999608

1600002K03Rik	1.302782	0.022423	0.999608
Jund	1.306884	0.014114	0.999608
Zfp628	1.314299	0.001256	0.999608
Aldh16a1	1.315987	0.002608	0.999608
Apoc4	1.316087	0.048755	0.999608
Junb	1.325326	0.009142	0.999608
Gm13192	1.337164	0.049675	0.999608
Pex14	1.344183	0.011234	0.999608
Ddah2	1.353519	0.040055	0.999608
Klhdc8b	1.354577	0.023777	0.999608
Crb3	1.370045	0.007396	0.999608
Pkmyt1	1.370049	0.001845	0.999608
BC029722	1.370873	0.014708	0.999608
Gnas	1.371347	0.026945	0.999608
Gm13123	1.376367	0.023429	0.999608
Tmem205	1.379633	0.026678	0.999608
Ccdc74a	1.389872	0.044443	0.999608
Ier5l	1.402111	0.012424	0.999608
Asic1	1.402717	0.011517	0.999608
Erf	1.402757	0.004417	0.999608
Tnfrsf12a	1.43068	0.0166	0.999608
Prr7	1.434546	0.016501	0.999608
Gm21887	1.43506	0.032824	0.999608
Ndufa12	1.438133	0.001159	0.999608
Gm45133	1.438207	0.029059	0.999608
Gm15247	1.447652	0.033789	0.999608
Gm10177	1.454321	0.007806	0.999608
H2-Bl	1.457391	0.041893	0.999608
Sssca1	1.458841	0.004668	0.999608
Rps18-ps3	1.463145	0.025886	0.999608
Gm10335	1.463307	0.006084	0.999608
Bbc3	1.464581	0.004686	0.999608
Gm10132	1.466278	0.007913	0.999608
Taf1c	1.468429	0.001328	0.999608
Tmem238	1.470978	0.009859	0.999608
Mir5125	1.493826	0.02788	0.999608
Lars2	1.501115	0.003095	0.999608
Tmem198	1.513704	0.044234	0.999608
Rasl10b	1.520815	0.008391	0.999608
Rpl7a-ps5	1.521025	0.00487	0.999608
Crlf2	1.521366	0.007833	0.999608
Abcg4	1.545395	0.017837	0.999608
Rac3	1.547892	0.019786	0.999608

Ndufaf8	1.550926	0.009002	0.999608
Gadd45gip1	1.551985	0.001089	0.999608
1700020L24Rik	1.567771	0.013943	0.999608
Chac1	1.573108	0.025586	0.999608
Mrps34	1.594007	0.003473	0.999608
Gm14321	1.595149	0.024432	0.999608
Gm10736	1.601848	0.007371	0.999608
Gm16418	1.606514	0.039266	0.999608
1110065P20Rik	1.617104	0.003383	0.999608
Rhbdd3	1.619109	0.001684	0.999608
3010003L21Rik	1.623695	0.022712	0.999608
AC122481.2	1.63612	0.041706	0.999608
Sh3bp1	1.63767	0.012706	0.999608
Gm49322	1.644631	0.040807	0.999608
Rnf26	1.650273	0.013661	0.999608
Tmem160	1.650351	0.000918	0.999608
Hs3st6	1.6728	0.02559	0.999608
Dohh	1.682044	0.001754	0.999608
Spata5l1	1.701354	0.002655	0.999608
Fbxl15	1.707889	0.000792	0.999608
Npff	1.715867	0.032205	0.999608
Snord49a	1.716059	0.029808	0.999608
CT010467.1	1.725445	0.001178	0.999608
Gdf15	1.753256	0.002053	0.999608
Camk2n2	1.800599	0.022856	0.999608
Lrfn1	1.818292	0.016997	0.999608
Comp	1.836162	0.029949	0.999608
Angptl8	1.837228	0.026281	0.999608
Ppp1r27	1.839014	0.046004	0.999608
Rhbdl1	1.860108	0.038488	0.999608
Ttll11	1.866042	0.009113	0.999608
Rpl28-ps3	1.885644	0.028893	0.999608
Nrtn	1.927077	0.021468	0.999608
Gm26917	1.993568	0.000507	0.999608
Rpl23a	1.995883	0.000106	0.775193
Gm10320	2.005106	0.021194	0.999608
Gm3756	2.072599	0.000527	0.999608
Gm6451	2.081516	0.036275	0.999608
Fam131c	2.089692	0.002518	0.999608
Gm10263	2.101578	0.004223	0.999608
Rtn4rl2	2.116567	0.01707	0.999608
Scand1	2.116622	0.000937	0.999608
Gm17111	2.116781	0.04271	0.999608

CAAA01118383.1	2.126004	0.000447	0.999608
Atp5k	2.146427	0.002047	0.999608
Gm5529	2.178068	0.024637	0.999608
Pdxp	2.182681	0.002405	0.999608
4930513N10Rik	2.193975	0.04751	0.999608
6330562C20Rik	2.276568	0.031316	0.999608
Nkpd1	2.290845	0.00946	0.999608
Snord17	2.335909	0.049251	0.999608
Gm47283	2.372556	0.000157	0.775193
Slc39a5	2.38547	0.018884	0.999608
Gm9908	2.387661	0.010998	0.999608
Ptp4a1	2.438816	0.000456	0.999608
Sema6c	2.44892	0.007774	0.999608
B330016D10Rik	2.467695	0.026847	0.999608
Amn	2.473677	0.037867	0.999608
Gm14685	2.541731	0.003444	0.999608
Cox20	2.568906	0.027107	0.999608
Gm11702	2.583666	0.000478	0.999608
Gm18584	2.584108	0.029224	0.999608
Gm43555	2.622678	0.004827	0.999608
Sdhaf1	2.629329	0.003163	0.999608
Gm20949	2.677706	0.030886	0.999608
Gm18194	2.730153	6.11E-06	0.07564
CAAA01147332.1	2.779057	0.000739	0.999608
Snord32a	2.842598	0.019754	0.999608
Gstp2	2.84414	0.001712	0.999608
Gm10709	2.91637	0.000188	0.776631
Gm10138	2.944872	0.024001	0.999608
Snora78	3.062667	0.012219	0.999608
Gm44765	3.072846	0.038918	0.999608
Gm28529	3.189062	0.020971	0.999608
Gm8210	3.214673	0.019479	0.999608
Gm7774	3.218698	0.015667	0.999608
Gm9523	3.259055	0.024245	0.999608
Rpl30-ps9	3.306424	0.020007	0.999608
Prr22	3.361978	0.005794	0.999608
Vwa1	3.363217	0.020014	0.999608
Gm16174	3.618832	0.045919	0.999608
Gm30025	3.625159	0.049118	0.999608
Gm44349	3.632976	0.006942	0.999608
Kcnh3	3.635038	0.043235	0.999608
Gm15889	3.882518	0.047155	0.999608
Gm23935	3.988602	0.008522	0.999608

4930413G21Rik	4.006016	0.012239	0.999608
Kcnip2	4.060935	0.040728	0.999608
Snora2b	4.116396	0.035489	0.999608
Ttll9	4.20095	0.024454	0.999608
Rdh12	4.281787	0.046235	0.999608
Ube4bos3	4.313474	0.018901	0.999608
Mir142b	4.406339	0.00612	0.999608
Mir23a	4.412769	0.012016	0.999608
Gm48699	4.451729	0.047829	0.999608
4930552P12Rik	4.565872	0.039594	0.999608
Cpt1b	4.642387	0.033621	0.999608
Gm11185	4.664314	0.029846	0.999608
A930002H24Rik	4.718115	0.028209	0.999608
Gm9887	4.73027	0.027765	0.999608
Spint3	4.764137	0.042874	0.999608
Cyp2u1	4.766404	0.024762	0.999608
Gpr20	4.870823	0.017204	0.999608
Gm38088	4.918876	0.003102	0.999608
Gm11290	5.005938	0.016643	0.999608
Gm11767	5.070983	0.011875	0.999608
Hsd11b2	5.085649	0.002187	0.999608
Actl10	5.112295	0.020735	0.999608
Mrc2	5.18678	0.007199	0.999608

Table S2. Downregulated and upregulated DEGs in second group.

gene	log2FoldChange	pvalue	padj
Gm8126	-5.37861	0.002298	1
Gm9484	-4.57339	0.023012	1
Gm42109	-4.55766	0.032822	1
4930505K14Rik	-4.42021	0.033266	1
Vmn1r-ps24	-4.27391	0.048011	1
Gm6850	-4.07579	0.022323	1
Drc1	-3.90327	0.028143	1
Gm37748	-3.9021	0.039407	1
4933427E11Rik	-3.89569	0.028833	1
6030445D17Rik	-3.67881	0.047337	1
Gm10044	-3.55218	0.01774	1
Gm43920	-3.3275	0.046687	1
Gm35439	-3.13829	0.012129	1
Epha1	-3.02935	0.049854	1
Gm13410	-2.93538	0.033447	1
Ifit1	-2.85105	0.046157	1

Gm5117	-2.8148	0.024662	1
Gm28530	-2.65494	0.006252	1
Lpar4	-2.63273	0.039259	1
Gm35147	-2.57315	0.039288	1
Got2-ps1	-2.56499	1.06E-05	0.001194
A430027C01Rik	-2.56109	0.020395	1
Gm38009	-2.55568	0.014492	1
Gm13339	-2.49198	2.76E-09	1.92E-06
Gm15027	-2.48261	0.04698	1
Gm7327	-2.48054	0.000497	0.017796
Gm14632	-2.4746	0.04925	1
Rps26-ps1	-2.44072	6.47E-11	7.78E-08
Rps12-ps10	-2.40502	7.03E-09	3.87E-06
Gm2541	-2.3367	7.46E-05	0.004812
Gm4221	-2.33293	0.027844	1
Gm15764	-2.29109	0.043205	1
Gm12430	-2.26344	0.000847	0.025316
Serpinb9c	-2.23998	0.017922	1
Gm7049	-2.15654	0.00014	0.007576
Gm6039	-2.13792	0.002208	0.047592
Gm9001	-2.10995	0.003285	0.061234
Gm17771	-2.10506	0.04476	1
Gm18537	-2.10382	0.011745	1
Gm6565	-2.10362	5.13E-06	0.000672
Gm4865	-2.09018	0.009953	1
Gm15801	-2.06254	5.15E-09	2.98E-06
Gm21378	-2.0542	9.00E-08	2.53E-05
Spef2	-2.03414	0.009133	0.112068
Il16	-2.03082	0.045564	1
Tes3-ps	-2.02273	0.00452	0.073742
Lyg2	-2.0172	0.032423	1
Rps6-ps2	-1.98689	0.045003	1
Gm4705	-1.9624	1.07E-05	0.001194
Gm3325	-1.95251	0.001957	0.044504
Gm8783	-1.94955	0.00078	0.024041
Rpl30-ps3	-1.93664	0.001727	0.041044
Steap2	-1.93616	0.032374	1
Gm9118	-1.92104	3.31E-08	1.15E-05
Gm8242	-1.91132	0.021264	0.179208
2310001H17Rik	-1.90199	0.044694	0.263675
Gm14057	-1.89795	0.042935	1
Zfp979	-1.89105	0.013149	1
Gm3150	-1.89008	0.041269	1

mt-Ts2	-1.88858	0.00038	0.015171
Gm48027	-1.87195	0.011801	0.12878
Gm36189	-1.84411	0.036566	1
Gm6363	-1.8107	3.81E-08	1.26E-05
Rpl36-ps2	-1.78434	0.035748	1
Mx1	-1.77529	0.049934	1
Gm4742	-1.76435	0.02985	0.212884
Gm6767	-1.75918	0.033039	0.226929
Gm10268	-1.75917	0.00946	0.114171
Zfp712	-1.74617	0.00438	0.072591
Fbxo47	-1.73273	0.037337	1
2310040G24Rik	-1.71689	0.010278	0.121375
Gm43197	-1.70625	0.021971	0.182154
Gm6087	-1.69195	0.00211	0.046487
Gm14401	-1.68325	0.001561	0.038061
Gm6710	-1.67475	0.013679	0.140563
Gm10704	-1.66792	0.000466	0.017185
Gm1866	-1.66709	0.004338	0.072266
Gm1972	-1.66432	0.005717	0.08545
Rpl21-ps11	-1.65815	4.96E-07	9.78E-05
9230112D13Rik	-1.65715	0.045509	1
Gm8318	-1.60392	0.005454	0.083126
Zfp759	-1.60126	0.010532	0.122959
Gm42585	-1.59532	0.049881	0.278258
Gm9790	-1.59148	0.03279	1
Gm13147	-1.5902	0.009026	0.111267
Tnip3	-1.55665	0.006184	0.089108
Zfp97	-1.55224	0.012839	0.135626
Gm6741	-1.54771	0.034187	0.231211
Gm5844	-1.54093	0.027966	0.204524
6430511E19Rik	-1.5372	0.014727	0.146653
Gm48990	-1.52371	0.001081	0.029712
Arhgef37	-1.51247	0.016575	0.157104
Gm19510	-1.51147	0.028208	0.205711
Ifi207	-1.50846	0.024978	0.193235
Zfp78	-1.50694	0.017657	0.161141
Ap4b1	-1.49404	0.004115	0.069806
Gm10254	-1.4908	0.00074	0.023193
Has2os	-1.48816	0.004571	0.074031
Gm15542	-1.48814	1.38E-05	0.001428
4930558J18Rik	-1.47581	0.018203	0.16331
Gm5791	-1.46872	0.027294	0.201281
Zfp931	-1.46431	0.000536	0.018789

Gm14287	-1.45372	3.73E-06	0.000525
Gm29787	-1.45014	4.84E-06	0.000646
Gm18541	-1.44513	0.007599	0.100725
Hmgn2-ps1	-1.44093	0.048803	0.275494
1300002E11Rik	-1.43942	0.00064	0.021025
5430414B19Rik	-1.41333	0.034545	0.232028
Carnmt1	-1.40331	0.000262	0.011715
Gbp7	-1.39595	0.005928	0.086785
2610008E11Rik	-1.39223	0.000166	0.008514
Gm4130	-1.39216	0.000124	0.006906
Sumo2	-1.38803	0.000208	0.010045
Gm4950	-1.38711	1.09E-05	0.00121
Rps15a-ps4	-1.37044	0.021585	0.180443
AC122821.1	-1.3703	0.03874	0.246482
Atm	-1.36634	0.010798	0.124021
Ndufs6b	-1.36376	0.026502	0.199068
Hspd1-ps3	-1.36007	0.00026	0.01169
Zfp937	-1.35172	0.045184	0.265379
Gimap9	-1.34651	0.002959	0.057004
Gm13341	-1.32897	0.008617	0.108652
Zfp52	-1.32693	4.22E-06	0.000575
Gm16581	-1.32626	0.0085	0.107918
Ap3s1-ps1	-1.31302	0.012119	0.130723
Rps15a-ps3	-1.31064	0.007802	0.102387
Gm42892	-1.31059	0.044184	0.262778
Tcea1	-1.30681	0.000259	0.01169
mt-Th	-1.30486	0.019944	0.172037
Eya4	-1.30262	0.048728	0.275267
Reps2	-1.29751	0.034104	0.231119
Gm18959	-1.29331	0.037191	0.242113
Sacm1l	-1.29062	0.004241	0.071033
Gm14117	-1.28612	0.021106	0.178866
Gm43429	-1.28383	0.039741	0.250193
Rpl31-ps13	-1.28069	0.039193	0.247937
Itgb3bp	-1.27779	6.57E-05	0.00445
Il5ra	-1.27066	0.044687	0.263675
A430033K04Rik	-1.27039	0.028893	0.208762
Ppargc1a	-1.26947	0.003657	0.065476
Ywhaq-ps3	-1.26861	0.02918	0.210376
Glud-ps	-1.26714	0.021588	0.180443
Gm32014	-1.26519	0.036031	0.238541
Rpl17-ps3	-1.26269	1.94E-05	0.001802
Zfp960	-1.26099	0.009061	0.111593

Gm16433	-1.25491	0.026532	0.199068
Cxcl5	-1.25132	0.021209	0.178866
AC129328.1	-1.24713	0.000839	0.0252
Stxbp3-ps	-1.24674	0.021636	0.180641
Gm31793	-1.24526	0.024096	0.190107
Zfp53	-1.24362	0.000158	0.008228
Ttpa	-1.23213	0.046401	0.268705
Gm7879	-1.22976	0.013477	0.139515
Gm45871	-1.22943	0.017554	0.160541
Dand5	-1.22643	0.033635	0.22947
Gm4332	-1.21724	2.24E-07	5.37E-05
Gm8121	-1.20784	0.031954	0.222597
Ccng2	-1.19164	0.019156	0.167534
AU041133	-1.18981	0.042332	0.257561
Zfp958	-1.18433	0.003759	0.066597
Gm12693	-1.17447	0.000311	0.013399
Gm6563	-1.17394	0.003829	0.067295
AC098880.2	-1.16746	0.000486	0.017543
Zfp808	-1.16656	0.011001	0.12503
Gm14326	-1.16204	0.002732	0.05397
Ap3s1	-1.15876	1.99E-07	4.88E-05
Cmc1	-1.15714	3.18E-05	0.002565
Zfp51	-1.15683	0.000663	0.021573
Lysmd1	-1.1563	0.017469	0.160318
Gm3365	-1.15379	0.0044	0.07261
Gm26981	-1.1504	0.035823	0.238252
Uchl4	-1.15036	0.038616	0.245934
Rpe	-1.15027	0.017047	0.158595
AC154457.3	-1.14848	0.000857	0.0254
Cops2	-1.14687	1.84E-06	0.000304
Gm5577	-1.14284	0.024864	0.193056
Gm8451	-1.1423	0.002359	0.049212
Ifi203	-1.13875	0.038616	0.245934
Chac2	-1.13618	0.011051	0.12503
Ldha-ps2	-1.13554	0.003934	0.068158
B130055M24Rik	-1.13311	0.005945	0.086811
Sec23a	-1.13112	0.005687	0.08521
Stk26	-1.1311	0.000762	0.023535
Zfp54	-1.12999	0.001994	0.044893
Gm13544	-1.12704	0.017425	0.160243
Nab1	-1.12562	0.000332	0.013971
Rab6a	-1.12486	1.01E-06	0.000182
Serpinb5	-1.12377	0.013778	0.141085

Dynlt3	-1.12155	0.000381	0.015171
Tbc1d8b	-1.10272	1.18E-05	0.001273
Gm7099	-1.10036	0.024181	0.1902
Ppp1cb	-1.0974	0.004937	0.078361
Gm2981	-1.09424	0.035971	0.238462
Cpne3	-1.09357	0.00288	0.055879
Tcea1-ps1	-1.09087	0.023923	0.189418
Zfp938	-1.08965	0.004013	0.068873
Phf11d	-1.08916	0.012309	0.131608
Mastl	-1.08806	0.000248	0.011362
Zfp458	-1.08745	0.018106	0.16325
Thumpd3	-1.08164	4.91E-05	0.003596
Zfp345	-1.08085	0.012105	0.130723
Gm14409	-1.08025	0.005923	0.086785
Phf6	-1.07442	0.032187	0.223751
Gmpr2	-1.07359	0.007256	0.098051
Gm11216	-1.06932	0.035155	0.235329
Sh3bgrl	-1.06863	0.026453	0.199068
Zfp229	-1.06859	0.005219	0.081138
Hmmr	-1.06687	0.00756	0.100505
5430403G16Rik	-1.06574	6.90E-05	0.004538
Lrrcc1	-1.06445	0.002087	0.046351
Scml4	-1.0642	0.014153	0.143768
Tnfsf4	-1.06372	0.045459	0.265737
Rps12	-1.06363	4.42E-07	9.18E-05
Gm12435	-1.06314	0.044911	0.264248
Gm10250	-1.06095	0.000223	0.010473
Gm7072	-1.05778	0.000744	0.023193
Gm5835	-1.05732	0.022314	0.183141
Gm13136	-1.05605	0.044301	0.26313
Gfpt1	-1.05495	0.000252	0.011502
2010315B03Rik	-1.05467	0.024354	0.191117
Gm1818	-1.05333	0.003549	0.064154
Plscr4	-1.05232	0.01668	0.15737
9430018G01Rik	-1.05113	0.033949	0.230543
Zfp994	-1.04995	0.020412	0.175046
Sub1	-1.04809	0.000216	0.010276
Gm10243	-1.04616	0.024807	0.192726
Caap1	-1.04577	0.022242	0.182863
Zfp955a	-1.04555	0.018672	0.165826
Zfp981	-1.04244	0.048951	0.275621
Atg4c	-1.04036	0.000212	0.010169
Rsbn1	-1.03602	0.000958	0.027391

Gm8991	-1.03479	0.001151	0.030984
Esco2	-1.03343	0.005723	0.08545
Rpl6l	-1.03223	2.29E-05	0.002059
Polr2k	-1.03223	0.003903	0.068142
Gm39469	-1.02984	0.0394	0.248694
Ugt2b1	-1.02752	0.036313	0.239221
Ap3m1	-1.02653	0.000687	0.022033
Gm3531	-1.02616	0.000835	0.025165
Gm5611	-1.02492	0.01102	0.12503
Acbd5	-1.02296	1.53E-05	0.001559
Rp2	-1.02197	1.65E-05	0.001629
Magoh	-1.0214	0.000747	0.023227
Stag2	-1.02104	0.00486	0.077485
Zbtb26	-1.02101	0.007431	0.099785
Txndc17	-1.01648	0.000117	0.006642
Tmlhe	-1.01501	0.000119	0.006752
4930522L14Rik	-1.01421	0.003182	0.05981
Fundc1	-1.01193	0.01106	0.125034
Ptbp3	-1.0115	0.007783	0.102234
Rora	-1.00981	0.037336	0.242387
Nostrin	-1.00717	2.96E-05	0.002426
Zfp60	-1.00534	0.047944	0.273739
Ccdc122	-1.00521	0.036616	0.240137
Tmem69	-1.00469	0.045289	0.265438
Zfp961	-1.00173	0.049405	0.276903
Tmem109	1.002516	3.83E-05	0.002975
Vamp2	1.002651	0.00295	0.056977
Setd4	1.004871	0.018598	0.165728
Dynlt1c	1.005025	0.006017	0.087759
Tsen54	1.008441	0.000116	0.006612
Nabp2	1.009632	0.004147	0.070164
Pear1	1.009873	0.036638	0.240162
Slc9a5	1.010304	0.001327	0.034183
Pcbp4	1.01051	0.000121	0.006774
Tecr	1.01179	0.000385	0.015188
Gm15387	1.012657	0.002111	0.046487
Tssc4	1.012913	0.000116	0.006612
Tmc6	1.013394	0.002409	0.049908
Tmem171	1.016033	0.029301	0.210658
Mon1a	1.016429	0.001394	0.035371
Qpctl	1.01673	0.001459	0.0366
Sh3tc2	1.017887	0.000533	0.018729
Efna4	1.018436	0.002537	0.051718

Tex264	1.018551	0.000205	0.009983
Gnb2	1.018672	0.000556	0.019306
Aars2	1.020121	9.19E-05	0.005703
Rxrb	1.021441	7.71E-05	0.004922
Cd82	1.021821	0.044694	0.263675
Zdhhc8	1.021935	0.00011	0.006442
Neurl1a	1.021945	0.044757	0.263926
Adap1	1.023358	0.013305	0.138255
Acad12	1.024085	0.023643	0.187966
Grasp	1.024122	0.023546	0.187952
Zfp512b	1.02434	0.000814	0.024846
Gm10269	1.025074	0.037184	0.242113
Mydgf	1.025658	4.04E-06	0.000556
Scube3	1.026382	0.020672	0.176133
Gm16001	1.026728	0.042486	0.257907
Fbxw4	1.026746	0.003237	0.060614
Plbd2	1.027048	0.000431	0.016324
Abcd1	1.028196	0.011199	0.125952
Rexo1	1.030732	0.003293	0.061234
B3gat3	1.030872	0.004404	0.07261
Epb41l4aos	1.031168	0.000743	0.023193
Rnf215	1.031255	0.004513	0.073742
Manbal	1.031464	4.95E-05	0.003596
Coq8b	1.032122	0.014552	0.145579
2410002F23Rik	1.03571	8.15E-05	0.00515
Oaz1	1.036404	0.000101	0.0062
Adgra2	1.036707	0.003398	0.062547
Plppr2	1.036794	0.047402	0.272239
Prrg2	1.037439	0.011764	0.128478
Snrnp35	1.03958	0.002636	0.053012
Klf4	1.040724	0.026151	0.197833
Rpl28-ps1	1.041067	0.003195	0.059968
Pgls	1.042739	0.000363	0.014854
Rps29	1.043242	0.001532	0.03764
Hyal2	1.04359	7.78E-05	0.004945
Ampd2	1.043955	0.000365	0.01487
Pias4	1.044246	0.005253	0.08145
Hdac7	1.044314	0.000451	0.016927
Tmem258	1.045025	0.000917	0.02656
Zfp428	1.045364	0.049908	0.278258
Ccdc12	1.047314	0.000385	0.015188
Unc5b	1.052541	0.000142	0.007605
Adgrl1	1.05314	0.000673	0.021755

Notum	1.054322	0.021587	0.180443
Sac3d1	1.055012	0.000465	0.017185
Arl8a	1.056664	0.032987	0.226808
Atg4d	1.058018	1.39E-05	0.001428
Gigyf1	1.059013	0.005009	0.079077
1-Sep	1.061945	0.016505	0.156878
Syvn1	1.06277	0.032557	0.225376
Ltbr	1.062998	0.001609	0.039012
Ppp1r14b	1.063055	4.86E-05	0.003596
Spns2	1.063104	0.012597	0.133708
Mafg	1.06498	0.002476	0.050806
Junb	1.065116	0.001268	0.033055
Slc1a5	1.065173	3.51E-05	0.002759
Abtb2	1.066752	0.032582	0.225433
Ccdc9	1.067283	0.006324	0.090539
Rps10	1.067689	0.030848	0.217482
Hmcn2	1.068135	0.00529	0.081675
Ankrd13b	1.068612	0.001857	0.042999
Arsa	1.069045	0.018183	0.16325
Scamp3	1.06914	0.001125	0.030531
Dgat2	1.069325	0.028121	0.205314
Atp5d	1.069925	0.000468	0.017212
Zyx	1.071603	0.000102	0.0062
Ephb2	1.071645	0.038203	0.244602
Atxn7l3	1.072835	5.28E-05	0.003753
Asb6	1.073506	1.88E-06	0.000307
Entpd2	1.074115	2.94E-05	0.002426
Tbkbp1	1.076491	0.007486	0.09992
Nlrx1	1.076822	0.001628	0.039395
Dbn1	1.077307	0.000142	0.007605
Npr1	1.078025	0.005671	0.085062
Qsox1	1.079652	0.013277	0.138155
Dnajb5	1.079741	0.003064	0.058348
Wdr81	1.081629	0.034046	0.230962
Lzts2	1.082009	0.000976	0.027797
Fbxl6	1.082132	1.21E-05	0.001292
Gm28661	1.082158	0.030516	0.215995
Gm17201	1.08239	0.001081	0.029712
Rpl21-ps10	1.082541	0.014162	0.143768
Rps10-ps2	1.086028	3.91E-06	0.000544
Map2k7	1.086505	0.00315	0.059548
Dgcr2	1.086736	9.85E-05	0.006083
Sars2	1.087682	0.007189	0.097333

Tmsb10	1.088565	0.003421	0.062788
Zfp385a	1.089926	0.001688	0.040496
Olfml3	1.090741	0.017299	0.159755
Adamts10	1.091377	0.025628	0.195994
Hmga1	1.091706	0.000285	0.012565
Col18a1	1.092475	0.005919	0.086785
Gm10819	1.092542	0.048507	0.274841
Igsf8	1.092775	0.03977	0.250193
Os9	1.093581	0.003381	0.062437
Sptbn4	1.094187	0.004199	0.07059
Gm9800	1.096452	0.010733	0.123755
Klf16	1.097484	0.002229	0.047981
Slc4a3	1.099396	0.028387	0.206456
Sbk1	1.099956	0.022725	0.184239
Hmga1b	1.100306	0.005766	0.085803
Npdc1	1.100483	0.000657	0.021449
Plk3	1.100917	1.24E-05	0.001294
Ano8	1.101678	0.000262	0.011715
Tomm40l	1.103329	0.009003	0.111094
Ftl1-ps1	1.103747	0.006367	0.090865
Rela	1.103866	8.72E-06	0.001029
Mien1	1.104038	0.03801	0.243952
Egr1	1.10523	0.037608	0.243024
H2afj	1.105495	0.000109	0.006409
Sppl2b	1.106087	0.000709	0.022478
Nectin1	1.106344	0.007736	0.101919
Rpl9-ps6	1.108588	1.58E-06	0.000264
Apol9b	1.109559	0.012281	0.131412
Tmem79	1.112208	0.005811	0.086288
Polr3gl	1.112314	0.001446	0.036477
Aspscr1	1.112615	1.24E-05	0.001294
Atp5g2	1.112962	0.000145	0.007622
Abca7	1.11305	0.006847	0.095249
Nr1h2	1.113616	0.002242	0.048175
Tesk1	1.114272	0.00333	0.06172
Prrc2a	1.114389	2.84E-05	0.002394
Tjap1	1.117483	0.000456	0.01706
Timp1	1.118046	0.015611	0.152027
Xylt2	1.118226	0.000886	0.026045
Zfp219	1.119836	0.000107	0.006392
Gm12231	1.121573	0.01801	0.162842
Ppdpf	1.123103	0.002616	0.052788
Scx	1.12376	5.23E-05	0.003739

Tsku	1.127132	2.74E-07	6.23E-05
2700081O15Rik	1.127767	0.000167	0.008514
Gm14567	1.128963	0.049994	0.278409
Gm11273	1.129007	0.000149	0.007804
Timm13	1.12913	0.002125	0.046723
Ctsf	1.129924	0.004342	0.072266
Mlf2	1.132608	6.89E-06	0.000835
Leng8	1.132678	0.030552	0.216138
Ddr1	1.133434	2.82E-06	0.000419
Kdm6b	1.134139	0.001854	0.042999
Cfap157	1.134408	0.031991	0.222626
Naglu	1.134751	0.00536	0.082262
Apoe	1.137353	0.001494	0.03703
Abhd14a	1.138581	0.00121	0.032107
Adam15	1.139246	0.000421	0.016154
Fuz	1.140601	0.035094	0.235058
Unk	1.140832	4.02E-05	0.003088
Lzts3	1.141361	0.001175	0.031381
Gpc1	1.141377	0.000655	0.02141
Chrd	1.141546	0.008485	0.107918
Jak3	1.142221	0.002757	0.05422
Btn1a1	1.143041	0.0407	0.251675
Tarbp2	1.143151	2.45E-06	0.000368
Igsf9	1.143619	2.06E-06	0.000324
Nek8	1.143868	0.001302	0.033848
Bok	1.143988	0.033306	0.227971
Mmp10	1.14447	0.029402	0.210715
Shkbp1	1.145607	0.000128	0.007062
Gm10241	1.14628	0.002625	0.052877
Atp13a1	1.146831	2.21E-05	0.002002
Sdsl	1.153033	0.037163	0.242113
Tlnrd1	1.153582	0.000252	0.011502
Fstl3	1.153597	0.044672	0.263675
Slc45a3	1.153666	0.003165	0.059667
Atf4	1.153855	0.002015	0.045136
Sox12	1.155622	2.32E-05	0.002069
Sptbn2	1.156553	0.030888	0.217482
Dennd6b	1.15827	2.61E-05	0.002256
Meis3	1.162599	0.007473	0.099852
Pld3	1.163238	0.007781	0.102234
Zdhhc1	1.163387	0.000109	0.006409
Atf6b	1.163508	7.02E-05	0.004564
Adgrg1	1.167662	0.037241	0.242315

6330403L08Rik	1.167696	0.00031	0.013399
Mogs	1.167779	1.62E-05	0.001621
Bcam	1.167983	6.52E-08	1.97E-05
Tmem158	1.174049	0.005469	0.083266
Glmp	1.174617	0.002849	0.055536
Rbm38	1.182878	0.000107	0.006392
Rpl28	1.184349	2.86E-05	0.002394
Nckap5l	1.184517	0.002147	0.046887
Abcb9	1.18529	7.04E-05	0.004564
Fzd8	1.189435	0.003037	0.058085
Adgrb2	1.190556	0.016785	0.157726
Cox16	1.19611	0.017519	0.160436
Gpaa1	1.196956	0.00033	0.013971
Polr2j	1.197302	0.000157	0.008179
Ino80b	1.197638	0.000312	0.013399
Tmub1	1.200033	0.002002	0.04499
Fignl2	1.202047	0.006738	0.094558
Stx1a	1.207293	9.75E-06	0.001137
Rnf126	1.207764	0.000236	0.010964
Erf	1.211463	0.008347	0.107098
Map1lc3a	1.213136	0.039119	0.247923
Slc12a4	1.215566	0.005056	0.079542
Tle2	1.22114	0.008051	0.104725
Serf2	1.221695	0.00619	0.089108
Asic1	1.223042	0.003948	0.068159
Il4ra	1.223563	0.010845	0.124166
Sp5	1.225936	1.17E-06	0.000206
Klf2	1.226329	0.011236	0.126042
Dlk2	1.226946	0.027924	0.204329
Chpf	1.228731	1.26E-08	5.86E-06
Atn1	1.228774	5.21E-06	0.000676
Cdk20	1.229952	0.008439	0.107746
Mgat1	1.233569	6.89E-05	0.004538
Egln2	1.234811	2.35E-06	0.000357
Atp13a2	1.235909	0.000947	0.02715
Ilvbl	1.239636	0.000675	0.021755
Foxp4	1.239826	1.97E-06	0.000314
Coil	1.241134	0.000856	0.0254
Rnaseh2c	1.2441	5.98E-05	0.004115
Gm9791	1.245433	0.001554	0.038023
Zfp703	1.246165	0.000243	0.011236
Fam129c	1.247393	0.000917	0.02656
Rnf39	1.251473	2.13E-05	0.001952

Nrbp2	1.251573	0.03716	0.242113
Fxyd5	1.251843	0.00098	0.027862
Bex3	1.251891	4.07E-07	8.81E-05
Zfp524	1.256011	0.006612	0.093146
Rpp25l	1.25706	0.028054	0.20494
Mief2	1.259111	0.006419	0.091307
Sphk1	1.259853	0.000383	0.015188
Slc26a10	1.260091	0.008344	0.107098
Dvl1	1.260828	0.002185	0.047414
Tyro3	1.261939	0.000795	0.024329
Lrrc73	1.262948	0.040371	0.251533
Hcfc1r1	1.26374	0.004067	0.06945
Mllt6	1.266757	0.00574	0.085618
Zfp444	1.267108	0.00013	0.007101
Rrp9	1.268304	0.008912	0.110686
Rnf225	1.270152	0.022159	0.182733
Fam219a	1.27342	0.001207	0.032106
Snhg20	1.273537	0.003045	0.058085
Megf8	1.273606	0.002352	0.049212
Mrps12	1.273884	0.000783	0.024056
Mfge8	1.274885	0.005117	0.080218
Faim2	1.275109	0.036614	0.240137
Saysd1	1.275372	1.64E-05	0.001628
2310011J03Rik	1.277846	0.001975	0.04478
Chst13	1.278087	0.004412	0.07261
2210011C24Rik	1.278288	0.010159	0.120406
Tpgs1	1.278642	0.000175	0.008786
Macrod1	1.278961	0.047979	0.273739
Col1a1	1.282097	0.034336	0.231389
Il17rc	1.283368	0.000106	0.006341
Cbarp	1.287319	0.001493	0.03703
Gpt	1.288005	0.033461	0.228522
Tsen34	1.29019	5.90E-05	0.004115
Ephb6	1.292328	4.30E-05	0.003267
Ier5l	1.294555	0.004606	0.0745
Aspg	1.296354	0.023509	0.187947
Hnrnpa0	1.296978	2.09E-08	7.69E-06
Rpl26	1.297918	2.37E-05	0.002106
Zfp579	1.300509	0.000271	0.012036
Tspan4	1.300589	4.45E-07	9.18E-05
Ccdc85b	1.300735	6.82E-05	0.004527
AC121151.1	1.300774	0.011574	0.127319
Slc9a1	1.302043	0.001593	0.038729

Nlgn2	1.304286	0.001458	0.0366
Rap1gap	1.305328	1.24E-05	0.001294
Srf	1.305782	0.001888	0.043467
Fbxl19	1.307601	0.000168	0.008514
Rai1	1.308615	3.50E-07	7.79E-05
Gm16184	1.314066	0.018036	0.162918
Rom1	1.315196	0.004197	0.07059
Gm10736	1.316109	0.012233	0.131182
Slc2a8	1.318327	0.014477	0.145387
Socs3	1.321833	0.005856	0.086408
Grik5	1.321846	0.001504	0.03707
Cacna1g	1.322505	0.028886	0.208762
Tor2a	1.323017	1.25E-07	3.38E-05
Zmynd15	1.326695	0.002853	0.055536
Abcc10	1.327008	4.63E-05	0.003478
Gpr137	1.330689	5.15E-05	0.00372
Cldn4	1.331285	0.033705	0.229828
Spag4	1.333894	0.000836	0.025165
Gm7336	1.334199	0.000707	0.02245
Mier2	1.334784	8.38E-06	0.001006
Ormdl3	1.335291	0.010133	0.120316
Gm14681	1.335981	5.73E-05	0.004046
Mlxipl	1.336009	3.97E-05	0.003069
Gm13123	1.338822	0.018549	0.165509
Wnt6	1.339008	0.000373	0.015028
Itm2c	1.339391	0.000129	0.007089
Nat6	1.342003	0.013951	0.1424
Slc39a3	1.342696	7.67E-07	0.000147
Tubb4b-ps1	1.342939	0.006841	0.095249
Rps28	1.343044	1.10E-05	0.00121
Gm17828	1.346276	0.032284	0.224187
Atf5	1.346755	2.48E-05	0.002159
Plod1	1.348255	0.016381	0.156411
Noxo1	1.352536	0.019801	0.171789
Cox6a1	1.352984	0.000105	0.006341
Lingo1	1.357419	0.001653	0.039869
Clcf1	1.357552	1.67E-05	0.001636
Setd1a	1.36006	0.003299	0.061234
Rce1	1.36081	1.30E-06	0.000223
Fbrs	1.362145	0.000921	0.026561
Myo15b	1.362292	0.006982	0.095718
Cdk3-ps	1.36348	0.01428	0.144496
Ltbp4	1.363737	0.000906	0.026392

Trappc6a	1.367235	0.000619	0.02054
Gm17251	1.369507	0.034554	0.232028
Mbd6	1.369678	0.001696	0.04061
Gm5854	1.374462	1.92E-05	0.001797
Spns1	1.374589	3.16E-05	0.002563
Gm28192	1.376382	0.018009	0.162842
C130050O18Rik	1.381446	0.022874	0.185111
Gm31513	1.382848	0.049332	0.276708
Dnd1	1.386594	0.024504	0.191385
Col27a1	1.39012	0.00516	0.080639
Gm13192	1.390542	0.009821	0.117346
Gm12960	1.395204	8.66E-09	4.58E-06
Gm8129	1.396321	5.22E-05	0.003739
DXBay18	1.400252	0.022121	0.182703
Ssbp4	1.40355	2.41E-05	0.002107
Gm10240	1.404898	0.043149	0.260053
Gm6566	1.406174	0.011482	0.12708
Pcnx3	1.412858	0.00034	0.014134
Tmem39b	1.413091	0.001375	0.035065
Otop2	1.41395	0.048145	0.273826
Lmtk3	1.414736	0.000887	0.026045
2900093K20Rik	1.41959	0.026933	0.199958
Rabac1	1.424103	1.86E-05	0.001768
Phf1	1.424184	0.019933	0.172037
Ogfod2	1.425067	0.005258	0.08145
Ctsd	1.425677	1.07E-05	0.001194
Gm11808	1.426945	2.38E-07	5.52E-05
Wfikkn1	1.428676	0.025473	0.195033
Azin2	1.428775	0.020233	0.17419
Iba57	1.435091	2.89E-05	0.002399
Hipk4	1.435384	0.001094	0.029989
Armc5	1.436367	4.23E-08	1.36E-05
Nr2f6	1.437841	4.55E-07	9.26E-05
Gm15590	1.440377	0.017602	0.160758
Gm28198	1.44365	0.039941	0.25051
Gm15122	1.447424	0.012582	0.133659
Ddah2	1.448582	0.023076	0.18617
Endog	1.448744	0.001726	0.041044
Klhdc8b	1.449931	0.005519	0.083734
Gm6627	1.450246	0.003407	0.06262
Gm18194	1.450676	0.017139	0.159058
Slc12a9	1.450801	3.71E-06	0.000525
Esrra	1.450961	3.12E-05	0.002543

Ttyh3	1.451261	1.29E-07	3.41E-05
Gtpbp6	1.452174	0.003927	0.068158
Gm6485	1.458673	0.000589	0.020049
Col5a3	1.459383	0.026613	0.199142
Epn1	1.460762	5.37E-07	0.000104
Rlbp1	1.463141	0.016684	0.15737
Pgap3	1.464236	0.001468	0.0366
Gm10443	1.474659	4.95E-07	9.78E-05
H2afx	1.475375	6.63E-08	1.97E-05
Ppfia3	1.476416	0.004955	0.078418
Tead3	1.479302	1.75E-05	0.001686
BC037034	1.482189	1.87E-05	0.001768
Adamtsl5	1.483956	0.000577	0.019818
Gm44432	1.484002	0.029334	0.210675
Inafm1	1.485233	0.003758	0.066597
Gm14321	1.486522	0.011167	0.125786
Gm45855	1.489344	4.27E-07	9.10E-05
Napsa	1.490071	0.040076	0.25074
A230083G16Rik	1.497407	0.008251	0.106379
Gm17112	1.49876	0.01874	0.165925
Rn7sk	1.505141	0.044833	0.264167
Maz	1.5072	0.000531	0.018729
Plpp2	1.50818	6.31E-11	7.78E-08
Sssca1	1.511485	0.000603	0.020277
Dus3l	1.512866	0.000227	0.010659
Aplp1	1.512879	0.040586	0.251675
Capn15	1.514444	3.28E-10	2.89E-07
Slc52a2	1.514629	2.93E-06	0.00043
Thbs3	1.515807	0.001164	0.03113
Edn1	1.516337	0.008432	0.107746
Grin2d	1.520787	0.000246	0.011346
Emc10	1.524091	1.85E-08	7.18E-06
Ankrd23	1.531214	0.040179	1
Zfp628	1.531533	1.15E-08	5.83E-06
Rps10-ps1	1.532883	8.53E-06	0.001015
Cdh24	1.535226	6.68E-05	0.00448
Tspo	1.536819	9.64E-07	0.000177
Gm3362	1.539868	0.000171	0.008624
Col16a1	1.542266	0.001688	0.040496
Tmem259	1.544213	1.49E-08	6.55E-06
Fbxo24	1.544515	0.017862	0.162118
Mtss1l	1.550915	0.024742	0.192516
Gm11703	1.553964	1.83E-05	0.001749

1810032O08Rik	1.561193	1.55E-08	6.60E-06
Osgin1	1.563574	0.017837	0.162003
Cldn3	1.564688	0.000524	0.018606
Atp6v0c	1.565399	0.00146	0.0366
Crlf1	1.565789	0.000109	0.006424
Plekha4	1.570751	0.013068	0.137068
Rac3	1.578036	0.000481	0.017419
Hist1h2br	1.578619	0.016764	0.157677
Ppp2r3d	1.580086	5.14E-06	0.000672
Lemd2	1.583673	0.000996	0.028004
Abcg4	1.588958	0.004814	0.077123
Atp6v0c-ps2	1.593187	0.030736	0.217089
Khk	1.597459	0.012065	0.130657
Psd	1.598873	0.002794	0.054779
Gpd1	1.599229	0.014601	0.145922
mt-Nd4l	1.607288	7.03E-05	0.004564
Aldh16a1	1.60988	1.78E-08	7.14E-06
Itga7	1.611238	3.22E-10	2.89E-07
Rps18-ps3	1.611747	0.001214	0.032148
Cyp21a1	1.613906	0.003386	0.062437
Gm4613	1.616743	0.036993	1
Mmp11	1.61729	0.000221	0.010444
Map3k10	1.62219	2.42E-08	8.63E-06
Apoc4	1.62957	0.000186	0.00926
Gm9844	1.629673	0.000597	0.020275
Taf1c	1.631631	1.75E-08	7.14E-06
Frs3	1.632622	0.007049	0.096223
Gnas	1.63387	0.001594	0.038729
Aqp8	1.636796	0.027111	0.200313
Trp53i13	1.637195	0.00409	0.069493
H1fx	1.638461	1.91E-06	0.000308
Oplah	1.639898	5.35E-06	0.000686
Cby3	1.646193	0.001261	0.033006
Arhgap33	1.648314	1.72E-05	0.001673
Snord65	1.651179	0.019738	1
Nradd	1.651278	8.16E-07	0.000154
Crb3	1.656095	1.62E-05	0.001621
Pde4c	1.657408	0.000191	0.009427
Nova2	1.657425	0.042901	1
Mfsd10	1.665462	9.81E-06	0.001137
Angptl8	1.670142	0.008128	0.10531
Dok7	1.675724	0.017072	0.158659
Lncppara	1.678643	0.013404	0.13893

Gm3788	1.680709	4.88E-05	0.003596
BC029722	1.681597	5.65E-06	0.000705
Slc35e4	1.68455	4.82E-06	0.000646
Hic1	1.689996	0.001902	0.043645
0610010K14Rik	1.693085	0.030922	0.217549
Chkb	1.695055	0.047951	0.273739
Ndufaf8	1.698088	0.000183	0.009103
Scpep1os	1.698997	0.028238	0.205711
Lbx2	1.700085	0.040583	0.251675
Hs3st6	1.704454	0.010593	0.123007
Kcnc3	1.721208	7.34E-08	2.11E-05
Vgf	1.735279	0.004467	0.073332
Npff	1.744686	0.034301	0.231266
Nme2	1.744899	0.006091	0.088364
Mir142hg	1.74603	0.006248	0.08965
Ptms	1.7511	9.80E-10	7.62E-07
Gm20605	1.754889	0.025161	0.193937
Zfp771	1.762101	6.66E-10	5.50E-07
Gm4149	1.773498	5.83E-05	0.004099
Egfl7	1.773523	0.00048	0.017419
Mrps34	1.774239	0.000128	0.007062
Pkmyt1	1.780522	3.02E-10	2.89E-07
Tnfrsf12a	1.781496	0.000442	0.016644
Dusp8	1.784629	6.37E-05	0.004336
Efna3	1.787598	0.000603	0.020277
Fcer1g	1.793779	0.024266	1
Gamt	1.803529	0.04337	1
Gadd45gip1	1.806415	9.66E-07	0.000177
Gm14058	1.813558	0.003867	0.067682
Jund	1.813914	1.40E-06	0.000237
Scp2-ps2	1.826099	0.02113	0.178866
Gm11221	1.826431	0.005841	0.086401
Gm15247	1.827363	0.001264	0.033006
1600002K03Rik	1.835245	6.68E-06	0.000818
Rpl7a-ps5	1.837214	4.71E-09	2.98E-06
Pex14	1.839839	5.12E-09	2.98E-06
Rps11-ps2	1.842105	0.01597	0.154495
Bbc3	1.84299	3.54E-07	7.79E-05
Rasl10b	1.846462	5.82E-06	0.000719
3010003L21Rik	1.850291	0.006886	0.095429
Dohh	1.85255	1.90E-08	7.18E-06
Siglecg	1.861609	0.003269	0.061022
Lrfn1	1.863395	0.001772	0.041725

1110065P20Rik	1.873011	1.81E-07	4.52E-05
Tmem238	1.879353	0.000257	0.011689
Gm15682	1.880705	1.24E-06	0.000215
Tmem198	1.884777	7.70E-05	0.004922
Nat14	1.886084	0.026538	0.199068
Chac1	1.888781	0.005162	0.080639
Gm10177	1.903146	1.44E-07	3.65E-05
Rhbdd3	1.905375	1.29E-08	5.86E-06
Gm45133	1.908703	1.05E-05	0.001194
Gm10132	1.909172	4.86E-08	1.53E-05
Dusp2	1.911015	0.008731	0.10978
Crlf2	1.920152	5.97E-05	0.004115
Rhbdl1	1.922894	0.007572	0.100565
Gm22009	1.923885	0.003581	0.064554
Slc16a8	1.924018	0.007127	0.096694
Camk2n2	1.933204	0.006762	0.094558
Tmem160	1.93588	3.90E-11	5.73E-08
1700020L24Rik	1.937896	1.26E-08	5.86E-06
Gm12749	1.945193	0.038689	1
Slc24a5	1.949556	0.005045	0.079459
Foxo6	1.951187	0.035319	1
Tmem205	1.952937	0.000114	0.006579
H2-Bl	1.962681	0.000996	0.028004
Gm38102	1.96687	0.013121	0.137068
Snord49a	1.966918	0.000471	0.017235
Mir5125	1.978159	0.000293	0.012826
Prr7	1.978227	1.42E-07	3.65E-05
Notch3	1.98422	0.024498	0.191385
Ttll11	1.986107	0.000734	0.023118
Cdv3-ps	2.00241	0.0154	1
Gm15491	2.008758	0.03773	0.243094
Ltb	2.016884	0.014871	1
Gm14685	2.019113	0.014371	1
Fbxl15	2.02171	1.11E-07	3.07E-05
Bloc1s1	2.035943	0.014734	1
B330016D10Rik	2.047187	0.043731	1
Rasal1	2.048134	0.040792	1
Rpl31-ps11	2.053845	0.004861	0.077485
Lars2	2.061051	6.67E-16	1.76E-12
4930516B21Rik	2.07815	0.025797	1
Sh3bp1	2.092742	3.62E-06	0.00052
Gdf15	2.096672	3.38E-08	1.15E-05
6720483E21Rik	2.101055	0.026451	1

Spata5l1	2.106545	6.71E-08	1.97E-05
Cnih2	2.107349	0.021964	0.182154
Rpl7a-ps3	2.122603	0.031725	1
Gm21887	2.127056	0.001407	0.035559
8430422M14Rik	2.137686	0.012261	1
Rnf26	2.139401	3.17E-06	0.00046
Gm25835	2.161062	0.000207	0.010045
Ndufa12	2.164661	1.06E-06	0.00019
Fam131c	2.166135	8.89E-05	0.005597
Nrtn	2.170627	0.000176	0.008801
Socs1	2.1744	0.001348	0.034652
Gm10335	2.181723	1.64E-10	1.80E-07
Gm43965	2.19051	0.023979	0.189637
Dact1	2.195604	0.037998	1
Slc22a18	2.197456	0.042545	1
Ilk	2.218947	0.0065	1
Mmp3	2.218986	0.012032	1
S1pr4	2.221074	0.029273	1
Dcdc2b	2.237152	0.047595	1
Gm49322	2.258946	8.97E-05	0.005621
Enho	2.259185	0.033433	1
Vkorc1	2.271923	0.00055	0.019179
6330562C20Rik	2.279114	0.016764	1
Snord55	2.294127	0.000671	0.021755
Gm2574	2.317932	0.022565	1
Rpl18-ps1	2.3193	0.041469	1
2210017I01Rik	2.321267	0.002343	0.049212
Gm6304	2.333731	0.014966	1
Gm10263	2.359521	5.54E-06	0.000704
Pdxp	2.373806	0.000143	0.007605
Ptp4a1	2.375209	4.37E-05	0.003303
Eif4ebp3	2.377912	0.026153	0.197833
Gm13205	2.404248	0.003961	0.068159
Nkpd1	2.427143	0.00213	0.046732
Rpl28-ps3	2.42807	0.000299	0.013028
Gm19810	2.43155	0.049175	0.276131
Gm43480	2.434557	0.048847	1
Rpl23a	2.434653	1.94E-16	6.40E-13
Il27	2.439817	0.018043	1
CT010467.1	2.446904	9.14E-12	1.51E-08
Gm3756	2.452329	2.14E-05	0.001953
Gm15526	2.459026	0.012389	1
Gm10382	2.469606	0.000424	0.016154

Snora62	2.480075	0.011576	1
Sdhaf1	2.507379	0.000907	0.026392
Fam69b	2.511738	0.037378	1
Il17d	2.52135	0.005324	1
Atp5k	2.523618	2.23E-06	0.000342
Amn	2.527855	0.016931	1
Ripor3	2.539333	0.020381	1
Gm20949	2.562064	0.021212	1
Gm36447	2.569536	0.036484	1
Scand1	2.611	5.19E-09	2.98E-06
Gm17111	2.623535	0.001993	0.044893
Trpm5	2.630987	0.017918	1
Gm43555	2.644261	0.000949	1
CAAA01118383.1	2.645722	9.03E-15	1.71E-11
Gm26694	2.65485	0.002875	1
Insyn1	2.689871	0.000699	0.022314
Tmem235	2.695329	0.030889	1
Gm49027	2.713019	0.039327	1
Cyp46a1	2.724845	0.030846	1
Kcp	2.73187	0.001838	0.042753
Gm14419	2.73279	0.001858	0.042999
Gm11702	2.751554	2.04E-09	1.49E-06
F2rl1	2.78454	0.030013	1
Gm10353	2.787498	0.02157	1
Tomm6os	2.795829	0.007931	1
Ceacam16	2.819563	0.030222	1
Gm26917	2.820008	3.72E-15	8.19E-12
Cox20	2.851644	0.006598	1
Rtn4rl2	2.890394	3.29E-05	0.002633
Gm9523	2.898053	0.036599	1
Slc39a5	2.917306	0.000143	0.007605
Mir7059	2.922079	0.016323	0.156085
Gm9908	2.967242	2.10E-05	0.001945
Gm7774	2.969254	0.019288	1
Gstp2	3.022711	0.000163	0.00841
Kazn	3.031877	0.031285	1
Cracr2b	3.036615	0.008411	1
Gm5855	3.042966	0.009872	1
1500002F19Rik	3.101668	0.021434	1
Slc35g3	3.105585	0.008755	1
Gm37509	3.126028	0.008334	1
Vwa1	3.158762	0.028795	1
Gm11946	3.168785	0.035688	1

CAAA01147332.1	3.170868	2.29E-07	5.41E-05
Gm20568	3.181273	0.044811	1
Gm6473	3.182193	0.006959	1
mt-Ty	3.241237	0.003708	1
1700018A04Rik	3.252954	0.012065	1
Snord32a	3.254189	0.001228	1
Gm22192	3.256519	0.037658	1
Sema6c	3.265285	6.67E-05	0.00448
Prr22	3.311728	0.007865	1
Gm25612	3.320641	0.036683	1
Gm10138	3.32464	0.00121	1
Gm44349	3.364593	0.001257	1
Gm47283	3.370697	2.19E-06	0.000341
Prtn3	3.389934	0.042279	1
Snora78	3.392882	0.000624	1
Lrfn4	3.415807	0.015963	1
Scarf1	3.419517	0.031946	1
Gad1	3.419615	0.025251	1
Gm12473	3.426187	0.002984	1
Gm22486	3.453199	0.022638	1
4930413G21Rik	3.503046	0.011587	1
Gm10709	3.529947	1.87E-16	6.40E-13
A930017K11Rik	3.534179	0.004665	1
Gm2420	3.574613	0.018075	1
Gm22980	3.639582	0.022422	1
Gm7079	3.711734	0.042376	1
Gm31881	3.716526	0.046947	1
Gm16174	3.720912	0.015588	1
Gm26175	3.754094	0.046993	1
Gm8210	3.763015	6.87E-21	9.07E-17
Tmem255b	3.7852	0.003096	0.058877
1810062O18Rik	3.799308	0.00031	1
Ache	3.808817	0.037973	1
Plekhb1	3.817524	0.034189	1
Mir142b	3.874119	0.006135	1
1110013H19Rik	3.905873	0.027543	1
Derl3	3.92806	0.008254	1
Gm28529	3.928875	0.000106	0.006341
6430573P05Rik	3.97128	0.029118	1
C4b	3.980359	0.022984	1
Gjc2	3.995956	0.024498	1
Snora2b	4.062238	0.019579	1
Kcnip2	4.092091	0.025603	1

Gm24325	4.172183	0.017415	1
B930094E09Rik	4.225479	0.039137	1
G630016G05Rik	4.234062	0.012721	1
Gm30025	4.270617	0.002338	1
AC117232.1	4.288097	0.04858	1
9430064I24Rik	4.299155	0.046144	1
Foxn1	4.314745	0.045022	1
Gm11767	4.333243	0.043732	1
Gm42814	4.357622	0.009026	1
Gm16156	4.439309	0.041193	1
Gm11824	4.442217	0.032042	1
Gm29482	4.466883	0.003133	1
Gm49437	4.497767	0.043016	1
Gm5560	4.514633	0.038306	1
Gm45246	4.527406	0.028988	1
Gm8055	4.573747	0.031801	1
Mir365-2	4.573747	0.031801	1
Spdef	4.590429	0.023452	1
Adora2b	4.593116	0.025254	1
Kcnh3	4.638083	0.000507	1
Mrc2	4.673681	0.017476	1
Mir23a	4.682405	0.001177	1
Gm13889	4.695844	0.028046	1
Aanat	4.735688	0.047294	1
Gm48699	4.781889	0.013216	1
Hist1h3h	4.781889	0.013216	1
Ptafr	4.784212	0.014931	1
Hsd11b2	4.7889	0.002812	1
Cpt1b	4.797819	0.015999	1
Gm23935	4.870359	3.23E-20	2.13E-16
4933408J17Rik	4.901473	0.036656	1
Gm17826	4.950025	0.037337	1
Magohb	5.13206	0.00805	1
Msi1	5.14223	0.006206	1
Mir7678	5.257613	0.008374	1

Table S3. Metabolite profiles in control and treated groups.

**NAME**	**Control 1**	**Control 2**	**Control 3**	**Control 4**	**Control 5**	**Treatment-1**	**Treatment-2**	**Treatment-3**	**Treatment-4**	**Treatment-5**
5-Amino-1-(5-Phospho-D-	1.6	5.6	1.3	6.5	1.5	1.2	4.0	2.3	2.9	7.6

ribosyl)imidazole-4-carboxamide	1E+03	4E+03	0E+04	8E+04	5E+03	2E+05	2E+04	9E+05	0E+05	0E+04
adenosine	3.59E+07	1.20E+07	6.21E+06	1.46E+07	1.73E+07	6.00E+08	4.09E+08	7.74E+07	1.11E+07	5.07E+08
CDP	8.02E+04	3.06E+05	5.80E+05	1.11E+05	4.90E+04	5.10E+06	1.07E+05	2.09E+06	4.80E+05	1.67E+06
CMP	7.84E+05	9.69E+05	5.58E+04	1.54E+06	1.24E+06	2.04E+07	1.21E+07	2.02E+06	9.26E+06	4.52E+06
CTP	2.13E+05	1.39E+04	7.27E+05	6.68E+03	1.63E+02	7.15E+04	7.33E+04	1.01E+06	4.05E+05	2.82E+05
cytidine	8.17E+06	5.77E+05	3.47E+06	1.06E+07	3.90E+06	4.04E+07	1.47E+06	4.39E+06	1.09E+07	3.22E+06
Deoxyinosine	1.38E+06	6.82E+05	1.82E+06	1.10E+06	6.28E+05	9.12E+06	3.45E+06	1.70E+06	8.56E+06	1.80E+06
Deoxyuridine	8.01E+05	1.03E+05	2.03E+06	4.72E+05	6.08E+05	2.45E+05	1.08E+05	6.90E+05	1.78E+06	1.73E+06
GDP	4.60E+05	6.74E+05	1.02E+06	1.13E+05	7.26E+05	2.51E+06	1.90E+06	9.43E+05	4.99E+06	2.43E+06
GMP	1.95E+05	6.42E+05	2.02E+05	8.31E+05	3.16E+06	1.02E+07	3.15E+07	1.22E+07	4.45E+07	1.53E+07
GTP	2.89E+05	2.35E+05	1.36E+06	2.37E+04	1.23E+05	8.10E+04	2.25E+05	2.59E+05	1.03E+06	1.04E+05
guanosine	6.1	7.0	4.1	1.1	2.0	1.6	5.8	6.0	1.3	1.6

	4E+05	3E+05	6E+05	9E+07	5E+06	8E+07	1E+06	6E+06	7E+07	6E+06
Hypoxanthine	1.33E+07	2.06E+07	3.00E+06	4.34E+07	1.87E+07	1.82E+08	3.06E+07	9.84E+07	3.28E+08	2.30E+07
inosine	2.03E+07	3.93E+07	1.90E+06	2.44E+08	2.80E+07	1.27E+08	3.40E+08	1.43E+07	6.04E+07	1.94E+07
orotate	3.81E+06	3.68E+06	1.77E+06	7.89E+06	9.20E+04	6.07E+05	2.26E+06	5.59E+06	1.02E+07	2.67E+06
Thymidine	6.05E+05	3.71E+05	2.46E+05	1.71E+06	1.11E+05	6.60E+05	6.82E+05	3.45E+05	7.05E+04	4.30E+05
UDP	5.12E+06	1.64E+06	2.99E+06	1.09E+06	1.54E+06	4.17E+07	1.56E+07	3.09E+07	2.31E+07	1.54E+07
UMP	1.44E+07	2.80E+06	6.66E+06	1.82E+07	1.84E+07	4.53E+07	3.00E+07	3.94E+07	3.13E+07	2.34E+07
uridine	2.64E+07	5.89E+06	2.22E+07	4.20E+07	5.89E+07	1.40E+08	6.56E+07	9.32E+07	2.45E+08	3.03E+07
UTP	1.06E+06	4.27E+05	1.42E+06	1.84E+05	3.68E+05	1.07E+06	2.08E+05	1.61E+06	2.04E+06	1.13E+06
(-)-Salsolinol/NN-dimethyldopaminequinone-1	1.37E+06	4.68E+05	6.60E+04	1.34E+06	6.38E+05	1.32E+06	2.42E+06	4.79E+05	1.49E+06	9.15E+04
(4-hydroxy-3-methoxyphenyl)acetaldehyde	2.55E+05	2.01E+05	2.18E+05	3.31E+05	1.26E+05	2.27E+05	1.08E+06	1.74E+04	4.50E+05	1.90E+05
(R)-carnitine	9.0	1.1	1.1	2.4	1.4	2.5	1.7	1.0	1.9	1.6

	1E+08	4E+09	3E+09	2E+08	5E+09	0E+08	0E+09	1E+09	2E+09	3E+08
(R)-mevalonate	8.59E+05	8.53E+05	3.15E+05	4.76E+04	7.74E+05	5.95E+05	7.87E+05	1.58E+06	1.32E+06	1.06E+06
(S)-dihydroorotate	1.72E+05	1.48E+05	3.93E+04	2.45E+05	1.07E+04	4.08E+05	5.02E+04	6.09E+05	9.41E+05	1.34E+05
11'-Carboxy-alpha-chromanol	3.40E+05	3.17E+05	3.01E+04	3.59E+05	4.28E+04	2.97E+06	3.52E+06	5.25E+05	1.83E+05	6.62E+04
12S-HHT	1.74E+05	1.75E+05	6.94E+04	1.45E+05	7.20E+04	2.45E+05	4.95E+05	2.58E+05	8.56E+05	2.17E+06
15(S)-Hydroxyeicosatrienoic acid	4.03E+04	9.38E+03	1.74E+05	1.64E+06	1.02E+05	2.68E+06	7.42E+06	7.56E+04	4.91E+06	1.37E+05
1-methylnicotinamide	3.35E+08	3.59E+08	2.82E+08	9.71E+08	8.25E+08	1.09E+08	1.12E+09	4.25E+08	8.67E+08	3.17E+08
1-methylpyrrolinium	4.38E+06	4.89E+05	4.23E+06	3.98E+06	3.52E+06	2.27E+07	2.61E+07	4.35E+06	3.12E+06	5.68E+06
2-aminoacrylic acid	2.97E+06	1.21E+06	4.25E+06	1.42E+06	2.29E+06	4.09E+06	6.78E+06	9.27E+05	6.27E+06	1.99E+05
2-aminomuconate	5.10E+05	1.62E+05	9.56E+04	1.36E+06	2.76E+05	8.58E+05	8.74E+05	4.51E+05	6.65E+05	2.16E+05
2-deoxycytidine	1.40E+06	1.46E+04	1.17E+06	2.20E+06	1.69E+05	4.34E+06	1.05E+06	2.25E+06	7.56E+05	9.56E+03
2-deoxy-D-ribose 5-phosphate/2-	3.4	1.6	1.3	2.0	6.1	1.0	1.2	1.2	1.3	3.1

Deoxy-D-ribose 1-phosphate	2E+05	0E+04	4E+05	9E+05	6E+04	8E+06	2E+06	9E+06	4E+06	1E+05
2-Hydroxyglutarate	1.37E+06	7.27E+06	1.06E+07	8.36E+06	1.72E+07	2.12E+07	3.12E+07	1.21E+07	8.55E+06	1.18E+06
2-keto-3-deoxy-D-glycero-D-galactononic acid 9-phosphate	4.33E+05	2.25E+05	7.54E+04	2.78E+04	5.28E+04	3.37E+05	3.81E+05	8.14E+05	6.49E+04	8.64E+04
2-keto-4-methylthiobutyrate	6.68E+02	8.44E+02	1.14E+04	2.30E+04	9.41E+04	7.02E+04	1.34E+04	2.73E+04	3.23E+04	2.59E+04
2-methylglutaconic acid	3.59E+05	6.59E+05	1.71E+04	7.83E+05	1.42E+06	1.60E+06	2.64E+03	8.23E+05	1.19E+06	5.41E+05
2-oxoglutarate(2-)	2.30E+06	6.23E+06	4.54E+05	2.13E+06	8.95E+06	1.92E+07	9.32E+06	2.01E+07	2.55E+07	2.03E+06
3(S)10(R)-OH-octadeca-6-trans-412-cis-trienoate	1.61E+05	3.61E+05	2.96E+05	1.62E+05	1.17E+05	1.73E+05	1.04E+06	1.04E+06	8.64E+05	3.52E+05
3(S)6(R)-dihydroxy-tetradec-8Z-enoate/3(S)6(S)-dihydroxy-tetradec-8Z-enoate	4.82E+05	1.10E+06	7.09E+05	3.47E+05	1.05E+06	9.17E+05	5.25E+05	1.65E+06	1.20E+06	1.42E+06
3-Deoxy-D-glycero-D-galacto-2-nonulosonic acid	1.64E+06	5.14E+05	8.24E+05	1.58E+06	2.00E+06	7.80E+05	1.83E+06	1.61E+06	1.65E+06	1.62E+06
3-hydroxyanthranilate	2.07E+05	3.36E+05	2.50E+05	3.27E+05	2.49E+05	1.81E+06	2.38E+06	5.93E+05	1.82E+06	4.64E+05
3-Hydroxybutyrylcarnitine	3.41E+07	2.56E+07	2.04E+06	1.06E+07	1.43E+07	4.17E+07	5.34E+07	3.87E+07	6.89E+07	1.24E+07
3-hydroxyhexadecanoylcarnitine	1.2	1.1	1.7	1.5	9.1	5.7	6.5	2.1	4.0	7.6

	0E+06	3E+06	4E+05	8E+06	7E+05	1E+06	0E+06	2E+06	2E+06	8E+05
3-hydroxyhexadecenoylcarnitine	2.21E+05	1.02E+05	6.19E+04	2.54E+04	6.73E+05	4.15E+05	9.20E+05	6.88E+05	5.10E+05	3.79E+05
3-hydroxy-isovaleryl carnitine	4.29E+06	1.69E+06	1.33E+05	2.50E+06	3.87E+06	3.14E+06	2.17E+06	7.71E+06	4.69E+06	6.66E+06
3-Hydroxy-N6N6N6-trimethyl-L-lysine	2.04E+06	1.33E+06	2.58E+06	1.66E+06	6.98E+05	1.20E+07	1.17E+07	4.13E+06	1.04E+07	3.98E+06
3-Keto-beta-D-galactose/L-Gulonolactone	3.00E+05	3.14E+05	3.42E+05	2.42E+05	4.85E+05	1.33E+06	2.75E+05	3.12E+05	1.01E+06	8.23E+05
3-methyl-2-oxobutanoate	6.64E+05	2.57E+05	4.31E+05	1.40E+06	1.46E+06	5.21E+06	4.29E+06	1.29E+06	2.98E+06	4.51E+06
3-methyl-2-oxopentanoate/4-methyl-2-oxopentanoate(ketoleucine)	2.30E+07	1.82E+07	2.97E+07	3.92E+07	5.94E+07	6.41E+07	2.62E+08	5.80E+04	5.30E+07	2.39E+07
3-oxo-10(R)-hydroxy-octadeca-6E8E12Z-trienoate/3-oxo-10(S)-hydroxy-octadeca-6E8E12Z-trienoate	1.63E+05	5.15E+05	1.60E+05	6.08E+05	5.37E+05	6.26E+06	1.43E+07	3.67E+06	4.44E+06	3.78E+06
3-oxo-6(R)-hydroxy-tetradec-8-cis-enoate/3-oxo-6(S)-hydroxy-tetradec-8Z-enoate	1.43E+03	3.32E+03	9.71E+04	1.98E+04	4.96E+04	3.63E+06	5.74E+06	2.86E+06	2.63E+06	3.37E+05
3-oxo-8(R)-hydroxy-hexadeca-6E10Z-dienoate/3-oxo-8(S)-hydroxy-hexadeca-6E10Z-dienoate	4.85E+03	1.66E+03	3.64E+03	5.52E+02	2.89E+04	9.60E+03	1.95E+05	6.05E+04	3.73E+03	1.37E+05
3-oxopropanoate	3.02E+06	1.98E+06	1.05E+06	2.89E+05	4.67E+06	3.86E+06	1.08E+07	9.73E+06	7.84E+06	1.11E+06
4(R)-hydroxy-dodec-6Z-	4.8	4.6	2.8	1.3	8.5	1.1	2.0	7.5	3.8	1.3

enoate/4(S)-hydroxy-dodec-6Z-enoate	7E+06	9E+05	8E+06	3E+06	7E+05	6E+07	9E+07	9E+06	9E+06	5E+06
4,6-Dihydroxyquinoline/quinoline-4,8-diol	6.17E+04	7.56E+04	2.20E+05	2.31E+05	4.69E+03	3.66E+05	5.52E+05	1.18E+05	1.22E+04	2.32E+04
4-hydroperoxy-2-nonenal	1.77E+06	1.99E+06	3.39E+06	2.04E+06	3.07E+06	1.96E+06	5.53E+05	1.19E+07	3.98E+06	9.75E+06
4-hydroxy-2-nonenal/3,4-epoxynonanal	9.88E+05	4.17E+05	1.22E+06	1.32E+06	1.65E+06	2.35E+06	4.59E+06	2.54E+05	8.41E+05	2.07E+06
4-hydroxybenzoate/Gentisate aldehyde	1.96E+06	2.14E+05	1.40E+06	2.91E+06	3.54E+06	1.67E+06	4.85E+06	1.91E+06	3.22E+06	3.80E+06
4-Imidazolone-5-propanoate	3.58E+06	2.28E+05	2.12E+06	1.54E+06	8.08E+05	2.95E+06	1.08E+07	8.04E+06	4.61E+06	8.91E+05
4-pyridoxate	3.56E+04	1.01E+04	2.45E+04	9.02E+04	2.82E+04	2.53E+03	3.36E+05	1.95E+05	1.96E+04	6.83E+04
5,6-dihydrothymine	3.06E+06	6.06E+05	1.35E+06	6.76E+05	1.80E+06	8.71E+06	2.15E+06	2.15E+07	6.44E+06	5.17E+06
5-Methoxyindoleacetate	1.20E+04	7.81E+04	1.33E+05	3.67E+05	2.18E+04	1.63E+05	4.29E+06	1.19E+06	4.56E+05	9.81E+04
5-methoxytryptophol	2.38E+05	9.01E+04	2.91E+03	1.74E+04	1.98E+05	1.17E+06	1.13E+06	1.03E+06	1.10E+06	4.90E+05
5-oxoproline/L-1-Pyrroline-3-hydroxy-5-carboxylate	6.81E+06	5.29E+06	1.63E+06	6.49E+07	1.27E+07	6.32E+07	5.06E+07	3.63E+06	4.53E+06	8.17E+06
6(S)-hydroxy-tetradeca-2E4E8Z-	1.4	1.5	1.1	1.6	2.0	8.5	1.5	4.9	9.2	4.9

trienoate/6(R)-hydroxy-tetradeca-2E4E8Z-trienoate	7E+06	6E+05	0E+05	8E+05	4E+05	4E+06	9E+07	2E+06	1E+05	8E+06
6-Phosphogluconic acid	3.01E+04	2.17E+03	1.63E+05	1.50E+04	2.08E+04	9.03E+05	3.15E+05	9.41E+05	2.42E+06	1.02E+05
7,9-dihydro-1H-purine-2688(3H)-trione	9.12E+06	1.34E+07	1.60E+07	2.54E+07	1.21E+07	2.39E+07	1.14E+07	5.84E+07	2.10E+07	2.66E+07
8,11,14-Eicosatrienoic acid (DGLA)	3.06E+06	4.97E+05	6.85E+05	1.95E+07	8.18E+05	2.80E+07	2.66E+07	2.82E+07	5.47E+06	5.15E+06
9(10)-EpOME/12(13)-EpOME	2.55E+05	2.45E+06	1.96E+06	7.14E+06	8.09E+05	9.80E+06	8.49E+06	2.90E+06	5.79E+06	7.53E+05
9,10-hydroxyoctadec-12(Z)-enoate/12,13-hydroxyoctadec-9(Z)-enoate	6.82E+05	1.32E+06	8.34E+05	1.82E+06	6.93E+05	2.28E+06	2.46E+05	3.18E+06	2.41E+06	9.56E+05
acetamidopropanal	1.00E+09	6.11E+08	9.54E+08	2.27E+08	2.41E+08	1.85E+08	1.49E+07	6.94E+08	1.35E+09	5.71E+07
acetoacetate	2.87E+06	3.64E+06	1.68E+06	1.32E+06	1.73E+06	2.25E+06	2.47E+06	3.59E+06	3.99E+06	1.05E+06
acetylcholine/4-(trimethylammonio)butanoate	1.03E+07	2.65E+07	1.30E+07	1.39E+07	6.09E+06	4.51E+07	2.61E+07	3.05E+07	1.62E+07	4.63E+07
adenine	3.86E+06	6.86E+06	8.73E+06	1.64E+06	1.49E+07	3.25E+07	6.45E+07	1.67E+07	4.03E+07	5.28E+07
adipic acid	1.73E+05	2.20E+05	4.88E+05	9.19E+05	2.75E+05	6.90E+05	9.66E+05	6.39E+05	3.82E+05	8.49E+04
ADP	8.6	6.0	1.4	2.2	2.6	1.8	1.1	3.3	4.8	2.8

	9E+06	7E+06	0E+07	1E+06	7E+06	2E+07	3E+06	4E+07	6E+07	8E+07
ADP-mannose/GDP-L-fucose/ADP alpha-D-glucoside	4.54E+04	8.68E+03	2.59E+05	3.42E+04	3.27E+04	1.28E+06	6.82E+04	7.97E+05	7.74E+05	6.64E+05
ADP-ribose	5.41E+05	3.27E+04	5.50E+05	1.05E+06	8.83E+05	1.90E+07	3.24E+06	9.63E+06	1.14E+07	3.06E+06
alpha-D-glucose	1.20E+05	5.01E+06	9.82E+06	3.00E+06	1.57E+07	4.07E+06	5.85E+06	1.79E+06	4.26E+06	6.62E+06
alpha-linolenyl carnitine/gamma-linolenyl carnitine	1.50E+05	1.17E+06	1.00E+06	1.55E+06	6.52E+05	3.30E+06	1.50E+06	5.04E+06	1.74E+06	3.67E+06
AMP	2.02E+07	1.79E+07	1.27E+07	1.97E+07	4.73E+07	1.48E+08	1.91E+08	1.65E+08	3.20E+08	1.15E+08
anthranilate	1.79E+06	8.64E+05	4.77E+06	5.60E+06	2.05E+06	8.12E+07	1.76E+07	5.25E+07	5.43E+07	7.11E+07
arachidonic acid	9.66E+06	1.94E+07	1.66E+07	6.63E+07	6.12E+07	4.43E+08	5.36E+08	5.04E+07	3.71E+08	2.94E+06
arginine	1.07E+07	2.58E+05	1.08E+07	5.27E+06	8.02E+05	3.63E+07	7.68E+06	1.87E+07	1.77E+07	5.73E+06
aspartate	9.44E+07	4.69E+07	2.16E+08	7.40E+07	1.59E+08	1.05E+08	9.54E+07	5.75E+07	5.97E+07	1.45E+08
ATP	5.06E+06	2.06E+06	8.68E+06	1.02E+05	1.34E+06	2.54E+07	6.69E+05	2.67E+07	6.27E+05	1.48E+07
Azelaic acid	1.0	4.5	1.0	4.1	3.1	2.4	4.7	7.3	4.6	7.1

	5E+07	0E+06	8E+07	5E+06	6E+06	0E+06	3E+05	7E+05	0E+06	6E+06
benzoate	1.96E+06	9.66E+05	3.15E+06	4.59E+06	5.00E+06	8.08E+06	1.44E+07	3.22E+06	5.64E+06	4.60E+06
beta-1-4-mannose-N-acetylglucosamine	2.48E+05	5.10E+05	5.13E+05	5.74E+05	5.95E+05	7.83E+05	1.49E+05	1.06E+06	1.35E+06	3.96E+05
beta-carboline	4.66E+02	8.52E+04	2.82E+04	1.43E+04	3.54E+04	4.11E+03	7.99E+03	8.69E+03	5.61E+03	7.21E+04
beta-D-Fructose 1,6-bisphosphate	4.45E+05	1.18E+05	7.03E+05	2.01E+05	2.77E+05	4.37E+06	2.65E+06	6.93E+06	6.21E+06	2.99E+06
beta-hydroxy-beta-methylbutyrate	9.53E+05	2.57E+05	8.58E+06	1.70E+06	2.57E+06	2.30E+06	1.58E+07	4.23E+06	1.77E+07	2.25E+06
bilirubin	3.59E+05	4.88E+04	4.74E+05	4.20E+05	3.55E+05	4.02E+06	1.03E+07	3.38E+06	3.04E+05	7.86E+05
Biotin	9.21E+05	1.64E+05	6.58E+05	8.30E+05	6.00E+05	1.01E+06	4.13E+05	3.44E+06	4.17E+06	2.01E+05
butyryl carnitine	8.17E+07	1.10E+08	1.15E+08	9.37E+07	1.98E+08	1.62E+08	2.07E+08	2.49E+08	1.35E+08	9.51E+07
C16H26O3(4)	1.15E+06	7.31E+05	3.68E+05	8.09E+05	3.97E+05	4.34E+05	8.75E+05	4.66E+05	2.18E+05	2.86E+05
C20H28O(5)	4.53E+03	2.26E+04	1.03E+05	6.76E+04	1.28E+04	2.96E+05	3.78E+05	8.86E+04	1.04E+05	1.34E+05
C20H28O2(7)	3.6	7.4	9.5	1.3	9.2	6.6	7.6	3.8	1.5	3.7

	6E+04	3E+04	9E+04	2E+05	8E+04	3E+04	5E+05	3E+04	1E+05	8E+04
C20H30O(4)	1.61E+04	1.60E+04	2.84E+04	1.02E+05	5.68E+04	1.37E+05	4.57E+06	3.03E+05	1.25E+06	3.70E+05
C20H30O2(4)	1.35E+06	9.51E+05	8.51E+04	6.58E+05	4.48E+06	1.81E+07	6.08E+06	4.12E+06	1.46E+06	2.17E+06
C20H30O3(6)	9.42E+03	8.42E+03	3.11E+03	1.49E+05	1.32E+03	1.70E+05	1.95E+05	2.68E+03	4.79E+04	3.32E+02
C20H32O3(12)	3.75E+04	3.12E+03	9.21E+03	1.01E+06	1.06E+04	4.36E+05	2.83E+06	9.45E+05	1.02E+06	1.60E+05
C27H42O4(3)	1.76E+05	1.71E+05	2.65E+05	7.85E+05	4.39E+05	1.38E+06	6.98E+05	7.34E+05	9.08E+05	6.78E+05
C27H44O3(5)	1.99E+04	3.53E+05	1.17E+05	5.04E+05	3.91E+05	2.51E+06	3.21E+06	2.28E+06	4.49E+05	1.41E+06
C3H4O2(3)	4.25E+06	2.77E+06	2.30E+06	4.94E+06	9.32E+06	8.19E+06	1.33E+07	1.18E+07	9.17E+06	3.25E+06
C4H6O3(3)	1.42E+07	1.11E+07	9.85E+06	6.14E+06	1.85E+07	3.08E+07	4.24E+07	1.78E+07	2.42E+07	4.73E+05
C5H10O5(6)	1.12E+05	2.42E+05	5.03E+04	1.55E+05	6.15E+05	4.82E+05	4.50E+05	2.04E+05	4.97E+05	2.47E+05
C6H11NO3(4)	5.46E+05	3.22E+06	1.45E+06	1.71E+06	3.16E+06	6.26E+06	1.17E+07	3.28E+06	3.70E+06	3.43E+06
C6H12O6(5)	9.0	1.6	1.6	2.3	6.6	3.8	6.9	9.9	3.8	2.9

	3E+07	9E+08	2E+08	2E+08	1E+07	6E+08	7E+08	4E+07	8E+08	9E+08
C9H13N5O3(4)	1.53E+05	1.58E+05	3.42E+05	1.29E+05	2.74E+05	1.69E+06	5.52E+05	6.84E+05	2.94E+05	5.84E+04
C9H8O3(3)	3.53E+06	2.28E+06	1.07E+07	6.84E+06	1.07E+07	6.68E+06	4.19E+07	9.94E+06	1.13E+07	2.45E+07
caprate	3.72E+06	1.72E+06	1.08E+07	2.61E+07	3.96E+07	8.87E+07	1.08E+08	3.82E+07	6.28E+07	2.71E+07
carnosine	6.36E+05	3.30E+05	2.68E+05	2.78E+04	1.12E+06	1.38E+06	3.91E+05	1.22E+06	2.44E+06	2.30E+05
CDPcholine	8.60E+06	3.31E+06	3.04E+06	3.10E+06	5.85E+06	4.87E+06	1.61E+07	1.80E+06	2.33E+07	4.61E+07
CDP-ethanolamine	2.14E+06	9.31E+05	3.54E+06	3.40E+05	5.71E+05	1.54E+07	4.88E+06	5.24E+06	9.68E+06	5.82E+06
cholesterol sulfate	2.27E+05	4.72E+05	9.95E+05	1.75E+07	8.07E+05	2.41E+06	4.72E+05	3.28E+05	9.74E+05	1.69E+05
choline	8.67E+08	1.24E+09	5.68E+08	6.37E+08	4.72E+08	1.81E+09	4.14E+09	2.23E+09	1.78E+09	1.56E+09
choline phosphate	7.41E+08	2.78E+07	2.95E+08	4.65E+08	3.84E+08	1.68E+08	6.40E+08	2.12E+09	4.02E+07	8.47E+08
cis-aconitate	1.18E+05	1.32E+06	2.90E+06	4.39E+05	2.49E+06	2.37E+07	1.90E+06	2.14E+06	6.28E+06	1.12E+07
citrate/isocitrate	1.5	1.0	2.7	1.5	1.4	2.6	1.0	2.8	1.3	8.7

	4E+06	6E+08	5E+08	8E+07	4E+08	1E+08	1E+09	8E+08	0E+09	4E+08
clupanodonic acid/docosa-4,7,10,13,16-pentaenoic acid	4.15E+06	1.48E+06	1.45E+06	3.31E+06	4.05E+05	3.97E+07	4.77E+07	2.67E+07	3.00E+06	1.38E+07
CMP-N-trimethyl-2-aminoethylphosphonate	4.80E+05	2.11E+05	1.93E+05	3.69E+05	3.05E+05	1.02E+06	1.24E+06	9.91E+05	1.22E+06	2.17E+05
coumarin	5.62E+05	3.45E+05	9.93E+05	2.73E+05	1.59E+06	4.05E+06	4.90E+06	6.00E+06	4.32E+06	1.50E+06
creatine	8.55E+08	2.46E+09	1.55E+09	1.08E+09	1.24E+09	2.22E+09	4.88E+08	2.07E+09	3.46E+09	4.52E+08
creatinine	4.71E+07	6.50E+07	3.02E+07	1.81E+05	2.01E+07	2.81E+07	1.25E+08	3.25E+07	2.61E+07	7.34E+06
cytosine	2.01E+07	4.56E+06	1.06E+07	1.47E+07	4.11E+06	5.43E+06	1.15E+07	8.26E+06	1.27E+06	5.60E+06
D-Alanyl-D-alanine	5.27E+05	7.01E+05	7.68E+05	1.72E+06	1.13E+06	1.18E+06	3.27E+05	1.18E+06	3.81E+05	1.33E+06
decanoyl carnitine	5.02E+06	1.23E+05	1.50E+06	3.80E+06	2.77E+06	4.52E+06	5.06E+06	1.01E+07	4.91E+06	3.70E+06
D-Erythrose 4-phosphate	6.65E+04	2.70E+04	8.82E+04	8.80E+04	1.15E+05	4.12E+05	8.72E+05	3.22E+04	4.44E+05	1.47E+05
D-Glucosamine	1.14E+05	1.71E+05	7.21E+05	1.06E+06	7.87E+05	5.66E+04	1.78E+05	6.14E+04	5.05E+02	5.64E+04
D-Glucosamine 6-phosphate	3.5	1.0	6.4	2.0	2.3	6.8	4.1	6.4	4.3	3.2

	4E+05	0E+06	4E+05	5E+06	5E+06	9E+06	3E+06	0E+06	4E+06	5E+06
D-Glucuronate 1-phosphate	2.99E+04	4.32E+04	3.09E+04	4.05E+03	2.27E+04	6.51E+05	5.39E+05	6.55E+03	5.15E+05	1.11E+05
D-glycerate	5.89E+05	3.85E+05	6.47E+05	1.22E+06	1.12E+06	9.39E+04	2.16E+06	9.14E+05	7.90E+05	6.45E+05
D-Glycerate 3-phosphate/D-Glycerate 2-phosphate	2.32E+05	2.61E+05	2.94E+05	6.40E+04	1.42E+06	5.90E+06	1.72E+07	1.12E+07	2.30E+06	2.52E+06
Dihydroxyacetone phosphate	5.99E+04	1.43E+06	9.92E+05	1.73E+06	1.92E+06	3.72E+06	6.57E+06	1.68E+06	1.30E+07	6.52E+06
dIMP	6.58E+05	4.51E+05	2.76E+05	3.96E+04	3.19E+05	6.42E+02	2.43E+05	1.26E+06	2.62E+05	7.57E+05
Diphosphate	2.86E+06	9.17E+05	2.81E+06	1.54E+06	2.16E+06	2.74E+07	1.11E+07	2.16E+07	1.04E+08	1.17E+07
docosahexaenoate(DHA)	1.18E+07	4.23E+06	7.60E+06	1.52E+07	1.20E+07	1.84E+07	8.79E+07	6.14E+07	5.57E+06	3.21E+07
docosanedioicacid	3.10E+04	2.16E+04	7.89E+02	5.98E+04	6.74E+04	7.51E+05	8.51E+04	2.22E+04	8.56E+04	2.37E+05
dopaminochrome	1.05E+06	5.58E+04	6.22E+05	8.40E+05	6.90E+05	2.99E+05	2.01E+05	6.26E+05	8.19E+05	5.73E+05
dUMP	6.36E+03	1.52E+04	9.63E+03	2.45E+04	2.74E+04	8.68E+05	4.33E+05	6.06E+04	2.07E+05	1.38E+05
Eicosenoic acid	1.9	2.9	5.9	4.5	4.4	1.1	1.7	1.8	1.1	7.6

	5E+06	3E+06	0E+06	3E+07	4E+06	9E+06	6E+06	7E+07	6E+07	5E+06
Elaidic carnitine/Vaccenyl carnitine	1.76E+07	1.06E+07	3.28E+07	3.88E+07	2.57E+07	2.36E+08	1.59E+07	1.19E+08	9.97E+07	6.41E+07
Ercalcitriol	4.31E+04	7.91E+03	3.37E+04	2.37E+04	2.65E+04	2.21E+05	5.22E+04	1.27E+05	9.37E+04	2.78E+04
fructoselysine 3-phosphate	2.78E+05	4.30E+05	2.36E+05	4.80E+05	1.28E+06	3.39E+05	1.63E+07	5.79E+06	6.65E+06	4.29E+05
fumarate	3.98E+07	2.41E+07	4.78E+07	3.14E+07	2.44E+07	8.60E+07	4.16E+07	1.90E+07	3.85E+07	3.47E+07
G6P/F6P	6.22E+06	1.96E+06	3.86E+06	4.42E+06	8.58E+05	2.04E+06	1.01E+07	1.60E+07	1.78E+07	1.28E+07
Galactosylglycerol	5.65E+04	7.05E+04	5.32E+03	3.37E+05	4.65E+04	4.60E+05	2.95E+05	2.71E+05	3.93E+05	2.07E+05
gama-L-glutamyl-L-alpha-aminobutyrate	1.82E+05	1.07E+05	1.38E+05	4.06E+05	2.31E+05	1.43E+05	2.85E+05	2.64E+05	5.95E+05	4.84E+04
GDP-D-mannose	6.79E+04	9.41E+04	2.30E+05	1.90E+04	4.72E+04	4.65E+05	4.38E+05	9.92E+05	1.35E+06	7.39E+05
glutaryl carnitine	6.94E+04	1.02E+02	4.70E+04	2.59E+04	7.70E+04	1.45E+05	9.13E+05	1.47E+04	2.53E+06	1.86E+06
glutathione	1.96E+07	1.28E+07	4.52E+07	1.33E+06	2.20E+08	3.90E+08	6.81E+08	4.45E+08	5.72E+08	1.82E+07
Glycerol 3-phosphate	6.6	1.7	4.3	4.0	6.0	7.8	1.9	2.7	7.3	1.0

	3E+07	0E+07	7E+07	7E+07	7E+07	7E+07	2E+07	1E+07	0E+07	6E+07
glycine	5.53E+07	2.80E+07	1.07E+07	1.07E+08	3.97E+07	2.17E+07	3.78E+07	1.54E+07	8.00E+06	2.75E+07
glycine betaine	6.58E+08	3.47E+08	7.34E+08	2.06E+08	1.77E+08	4.06E+07	9.05E+08	1.66E+08	6.19E+08	2.63E+08
Glycylleucine	1.15E+06	3.12E+05	3.26E+06	5.92E+05	1.36E+06	4.54E+06	1.67E+05	3.14E+06	4.69E+06	1.05E+06
Glycyl-phenylalanine	2.60E+04	1.80E+04	4.36E+04	3.64E+04	1.09E+05	1.75E+05	9.38E+04	3.07E+04	2.56E+05	6.04E+04
Glycylproline/L-Prolinylglycine	8.62E+05	7.32E+05	6.17E+05	1.40E+06	9.28E+05	3.07E+06	5.41E+05	9.24E+05	1.76E+06	1.64E+06
GSSG	6.38E+06	1.65E+07	2.27E+07	1.36E+06	2.57E+07	8.36E+07	5.49E+06	1.36E+08	3.58E+07	1.08E+08
Guanine	8.00E+05	1.60E+05	5.00E+05	6.41E+05	6.40E+05	7.93E+05	1.02E+06	3.79E+05	1.31E+06	4.58E+05
gulonate	2.58E+07	1.07E+07	3.67E+07	9.68E+06	1.68E+07	1.29E+08	5.11E+07	1.36E+08	2.17E+08	8.59E+07
heptadecanoyl carnitine	8.69E+04	8.63E+05	1.03E+06	5.11E+06	2.14E+06	1.21E+07	1.34E+07	2.68E+06	5.71E+06	3.30E+06
Hexadecanedioic acid	8.69E+04	1.08E+06	1.08E+06	5.45E+05	3.72E+02	1.71E+06	7.06E+05	3.73E+05	5.70E+05	1.25E+06
Hypotaurine	5.6	6.0	6.1	4.7	9.1	7.3	2.6	4.3	3.6	5.5

	7E+06	5E+06	3E+06	4E+06	4E+06	7E+06	6E+06	3E+06	4E+06	8E+06
isoputreanine/putreanine	3.86E+06	1.44E+06	4.38E+05	5.68E+06	9.80E+06	9.92E+06	5.18E+06	7.67E+06	1.03E+07	4.12E+06
itaconate/Mesaconate	8.35E+06	2.16E+06	7.54E+06	4.99E+05	5.48E+06	6.14E+06	3.31E+07	5.76E+06	2.00E+07	4.46E+06
kynurenate	3.15E+02	4.37E+04	3.37E+04	4.18E+04	7.86E+04	1.28E+05	1.51E+06	7.93E+04	9.87E+04	3.72E+03
L-4-hydroxyglutamate semialdehyde	8.65E+08	9.46E+08	9.08E+08	9.50E+08	7.91E+08	1.77E+09	5.59E+08	4.29E+08	6.97E+08	1.00E+09
lactate	2.77E+09	3.05E+09	4.46E+09	8.30E+09	1.68E+09	1.27E+10	8.65E+09	2.96E+09	6.71E+09	2.06E+09
L-alanine	1.03E+08	8.59E+07	1.78E+08	1.05E+08	1.13E+08	1.48E+08	1.55E+08	1.63E+08	2.05E+08	1.39E+08
L-Alanyl-L-leucine	5.08E+04	3.53E+05	1.22E+05	6.60E+05	3.92E+05	1.78E+06	1.20E+05	7.11E+05	9.08E+05	6.75E+05
L-arabinitol/xylitol/D-ribitol	2.99E+06	1.52E+06	7.69E+05	4.85E+06	1.46E+06	5.17E+06	8.44E+06	5.10E+06	3.34E+06	4.08E+06
L-asparagine	1.54E+07	1.47E+07	1.09E+07	2.77E+07	1.13E+07	7.79E+07	7.06E+07	3.41E+07	5.52E+07	2.97E+07
laurate	1.67E+07	2.94E+07	3.08E+07	4.37E+07	6.30E+07	1.10E+08	3.54E+07	4.63E+07	9.09E+07	6.40E+07
lauroyl carnitine	2.1	1.1	4.7	2.6	2.6	9.8	3.1	1.8	9.4	6.8

	6E+06	1E+06	2E+05	8E+05	5E+06	1E+06	9E+07	6E+07	7E+06	5E+06
L-cystathionine	1.52E+07	3.56E+06	3.40E+07	1.48E+07	2.20E+06	1.44E+08	7.46E+06	2.64E+07	1.88E+07	5.06E+07
L-cysteate	1.77E+06	1.43E+06	8.45E+05	1.35E+06	1.03E+06	8.09E+05	6.70E+05	8.85E+05	1.36E+05	6.91E+05
L-cysteine	1.01E+05	5.02E+04	3.86E+05	1.62E+04	2.14E+05	2.09E+05	3.03E+05	1.03E+05	7.19E+03	6.13E+03
L-cysteinylglycine	2.89E+06	1.44E+06	1.42E+06	8.46E+05	1.55E+06	1.96E+07	9.03E+05	4.42E+06	1.21E+07	1.22E+06
L-erythrulose	5.87E+06	1.01E+05	6.00E+06	3.51E+06	2.00E+06	1.30E+06	6.34E+05	2.34E+06	1.67E+06	1.20E+06
L-Fucose 1-phosphate	9.73E+05	5.26E+04	9.04E+05	1.28E+06	1.09E+05	8.18E+06	6.46E+04	1.74E+06	1.16E+06	2.33E+06
L-fucose/L-Fuculose	3.25E+04	6.72E+04	3.61E+04	4.55E+03	3.92E+04	6.24E+04	6.05E+04	1.15E+04	3.31E+04	5.27E+05
L-glutamate	1.06E+08	2.30E+08	7.30E+08	8.92E+08	9.33E+08	7.63E+08	1.59E+09	9.88E+08	2.10E+09	5.47E+08
L-glutamine	2.10E+08	1.69E+08	8.69E+07	4.39E+07	1.85E+08	2.39E+08	9.26E+07	7.52E+07	1.36E+08	1.82E+08
L-histidine	8.46E+07	4.90E+07	8.53E+07	9.48E+07	6.31E+07	1.31E+08	1.55E+08	4.91E+07	1.45E+08	6.79E+07
L-homocysteine	6.4	5.7	1.6	7.3	5.2	4.5	5.8	8.7	9.4	7.4

	1E+04	4E+04	3E+04	4E+03	7E+03	6E+04	5E+02	3E+03	6E+03	7E+02
L-Iditol/D-glucitol/galactitol	7.17E+05	2.76E+05	1.06E+06	5.45E+05	1.68E+06	1.30E+05	1.30E+06	3.52E+06	1.58E+06	1.36E+07
limonene/(+)-alpha-pinene	1.30E+04	3.50E+05	3.37E+05	3.05E+05	1.80E+05	2.62E+06	4.31E+06	6.01E+04	1.67E+06	2.68E+06
linoleic acid/linoelaidic acid (all trans C18:2)/octadecadienoate (n-C18:2)	3.00E+07	6.88E+07	9.77E+07	6.75E+08	6.26E+06	7.56E+07	2.29E+07	3.88E+08	7.24E+08	3.81E+08
Linoleyl carnitine/Linoelaidyl carnitine	3.20E+07	2.05E+07	2.84E+06	3.27E+07	2.63E+06	4.31E+07	3.97E+07	1.60E+07	1.45E+08	7.72E+07
L-isoleucine/L-leucine	1.64E+08	1.69E+08	2.18E+08	6.15E+07	9.80E+07	2.02E+07	1.08E+09	7.16E+08	1.24E+08	3.10E+08
L-kynurenine/Formyl-5-hydroxykynurenamine	1.33E+05	1.54E+05	2.63E+05	1.90E+05	4.80E+05	1.54E+06	9.62E+05	6.31E+05	1.07E+06	1.99E+05
L-methionine	3.72E+07	2.91E+07	8.34E+07	7.61E+06	4.46E+07	8.70E+07	2.83E+07	6.93E+07	1.11E+08	6.97E+07
L-palmitoylcarnitine	7.03E+07	2.65E+07	8.52E+06	1.02E+08	6.96E+07	5.14E+08	2.72E+08	1.57E+08	2.58E+08	1.96E+08
L-phenylalanine	1.00E+08	7.72E+07	9.31E+07	1.96E+08	2.31E+08	2.40E+08	6.29E+08	5.40E+07	2.94E+08	3.17E+08
L-proline	1.82E+08	9.59E+08	2.13E+08	1.19E+06	5.25E+08	1.10E+09	1.65E+09	6.93E+07	1.86E+09	1.01E+08
L-threonate	5.9	1.8	2.2	7.3	1.5	7.7	1.7	3.2	7.0	4.8

	5E+07	0E+07	4E+07	3E+07	1E+07	1E+07	3E+08	0E+07	7E+07	1E+07
L-threonine	7.29E+07	6.76E+07	5.18E+07	1.47E+08	8.25E+07	1.92E+07	1.00E+08	6.68E+07	1.49E+08	1.33E+08
L-tryptophan	4.90E+07	3.23E+07	6.02E+07	5.33E+07	1.41E+06	2.58E+08	1.30E+08	1.20E+08	1.34E+08	9.80E+07
L-tyrosine	2.73E+06	2.66E+07	3.12E+07	7.83E+07	5.50E+07	1.65E+08	3.18E+07	1.45E+07	1.45E+08	3.78E+07
L-xylonate/L-lyxonate	9.81E+05	8.95E+05	4.08E+06	2.90E+06	2.13E+06	2.70E+06	6.33E+06	5.06E+06	1.52E+07	6.17E+06
lysine	2.27E+07	1.11E+07	3.62E+07	3.92E+06	2.41E+07	4.92E+06	1.33E+08	8.33E+07	2.70E+07	2.68E+07
malate(2-)	2.82E+08	3.50E+08	2.28E+08	1.89E+08	3.31E+08	1.93E+08	6.49E+08	3.03E+08	1.98E+08	4.81E+08
margarate	2.23E+06	2.47E+07	1.86E+07	2.25E+07	7.78E+06	1.38E+07	2.88E+07	1.67E+07	3.02E+07	1.40E+07
Methionine sulfoxide	5.85E+05	7.61E+04	4.98E+05	2.43E+06	1.94E+06	3.31E+05	1.48E+04	1.27E+06	1.54E+06	1.24E+06
methyl indole-3-acetate	8.55E+04	4.63E+04	5.93E+04	4.10E+04	2.30E+05	4.63E+05	6.56E+05	4.27E+04	1.10E+05	1.51E+05
Methylimidazoleacetic acid	5.47E+06	2.45E+06	4.00E+06	1.31E+07	1.17E+07	1.27E+07	8.40E+06	1.41E+07	1.74E+06	7.65E+06
Methylisocitric acid/2-methylcitrate	1.4	2.1	5.8	6.5	2.1	1.6	5.0	4.0	6.6	1.2

	1E+05	8E+05	9E+05	7E+02	5E+03	4E+05	3E+05	1E+04	6E+05	2E+06
myo-inositol	2.04E+08	1.05E+08	1.80E+08	8.82E+07	3.16E+07	2.90E+08	8.99E+08	3.10E+08	6.61E+08	6.13E+08
myristate	9.55E+08	1.77E+09	5.06E+08	1.02E+09	1.21E+09	2.22E+09	1.45E+09	1.17E+09	1.58E+09	5.68E+08
N(2)-acetyl-L-ornithine	1.39E+06	1.06E+06	2.01E+05	1.63E+05	6.10E+04	3.30E+06	9.12E+05	4.76E+06	2.50E+06	7.09E+05
N(omega)-(L-Arginino)succinate	3.65E+05	3.26E+05	3.27E+05	4.02E+05	1.90E+05	2.95E+04	7.97E+05	2.87E+05	1.04E+06	2.35E+05
N1,N12-diacetylspermidine	2.62E+05	4.89E+03	3.67E+05	1.31E+04	4.37E+05	4.31E+06	7.25E+04	1.53E+05	4.92E+06	6.18E+05
N6N6N6-Trimethyl-L-lysine	4.49E+06	1.18E+07	3.06E+06	1.24E+07	1.77E+07	8.60E+07	7.14E+07	6.80E+07	5.24E+07	3.57E+07
N-acetyl-L-aspartate/2-Amino-3-oxoadipate	1.57E+08	4.37E+07	1.48E+07	4.26E+08	8.24E+06	1.83E+08	1.14E+08	8.58E+06	1.24E+08	2.56E+07
N-acetyl-L-glutamate	4.41E+06	1.60E+06	6.00E+06	4.11E+05	2.70E+06	8.23E+06	3.15E+06	1.08E+07	1.14E+07	6.30E+06
N-Acetylneuraminate 9-phosphate	9.70E+04	9.14E+04	2.99E+04	9.52E+04	4.79E+05	2.85E+04	1.22E+05	1.30E+06	4.99E+05	2.92E+05
N-acetylneuraminic acid	3.49E+06	8.53E+05	3.96E+06	8.63E+06	2.72E+06	1.09E+07	2.94E+06	3.81E+06	1.28E+07	7.98E+06
N-acetyl-seryl-aspartate	1.0	2.7	2.8	6.9	3.9	6.1	3.2	2.6	6.9	5.7

	2E+06	3E+05	8E+04	3E+05	0E+05	2E+05	8E+05	8E+05	1E+05	4E+05
NAD+	8.01E+06	8.44E+06	2.68E+06	1.41E+06	4.53E+06	1.35E+07	2.38E+07	6.49E+07	4.54E+07	3.40E+07
naphthalene	8.63E+04	4.33E+04	1.40E+04	7.23E+04	1.86E+05	6.43E+05	3.50E+05	8.38E+05	4.17E+05	7.75E+05
N-Carbamoyl-L-aspartate	3.57E+06	2.25E+06	3.80E+05	2.88E+06	1.41E+06	3.29E+06	1.16E+06	1.16E+06	4.46E+06	4.10E+05
N-Formimidoyl-L-glutamate	3.22E+03	2.79E+04	2.42E+05	3.64E+05	3.05E+05	5.24E+05	5.35E+05	3.07E+05	7.11E+05	2.42E+05
N-formylanthranilate/noradrenochrome	9.16E+04	4.51E+04	3.03E+05	8.15E+05	1.69E+05	1.79E+05	1.28E+05	2.87E+05	1.67E+05	1.94E+05
Nicotinamide	3.66E+07	1.32E+07	1.48E+08	8.72E+07	2.34E+08	1.09E+09	1.45E+08	4.39E+08	7.65E+08	1.26E+08
N-methyl-467-trihydroxy-1234-tetrahydroisoquinoline	4.50E+04	3.02E+03	2.87E+04	3.58E+04	6.16E+03	5.30E+05	1.51E+06	1.21E+06	5.73E+05	6.88E+05
N-Methylethanolamine phosphate	4.41E+05	1.17E+05	2.80E+05	3.18E+05	7.38E+05	9.18E+05	8.88E+04	3.57E+05	4.97E+05	2.19E+05
NN-dimethylglycine	1.98E+06	2.48E+04	5.22E+06	5.77E+06	4.56E+06	5.08E+06	4.47E+06	6.41E+06	4.61E+05	1.19E+06
nonanoate	4.13E+06	1.59E+07	7.48E+06	4.34E+07	2.00E+07	4.32E+06	9.86E+06	4.74E+06	2.05E+07	3.29E+07
N-phosphocreatinate	1.3	1.3	1.9	1.1	7.2	1.5	4.4	3.6	3.5	3.2

	7E+07	8E+07	0E+07	9E+07	5E+06	9E+06	4E+06	8E+06	2E+07	1E+07
N-Ribosylnicotinamide	5.33E+05	9.87E+05	1.17E+06	9.34E+05	8.07E+05	3.78E+05	6.77E+04	4.38E+04	6.59E+05	9.93E+05
O-acetylcarnitine	7.40E+08	7.23E+08	5.75E+08	5.37E+08	6.87E+08	1.92E+09	8.21E+08	2.11E+08	5.41E+08	9.07E+08
octanoyl carnitine/L-Octanoylcarnitine	1.00E+06	5.64E+06	2.18E+06	2.46E+06	2.74E+06	7.48E+06	1.09E+07	1.80E+07	8.27E+06	8.71E+06
oleic acid/elaidate/trans-vaccenate	2.37E+08	5.42E+07	1.65E+07	6.36E+08	1.10E+08	1.11E+09	7.54E+08	2.19E+07	5.56E+08	3.93E+08
omega hydroxy dodecanoate (n-C12:0)	2.33E+06	1.83E+06	1.93E+06	1.94E+06	1.15E+06	4.29E+06	1.56E+07	1.54E+05	3.85E+06	4.28E+06
omega hydroxy hexadecanoate (n-C16:0)	2.09E+06	1.12E+07	1.06E+07	2.44E+07	6.88E+06	5.63E+07	2.01E+07	1.30E+07	6.87E+06	1.36E+07
omega hydroxy tetradecanoate (n-C14:0)	1.20E+06	4.92E+05	7.99E+05	1.53E+06	4.20E+05	4.99E+06	1.56E+06	4.41E+05	4.08E+06	3.30E+06
Ornithine	2.42E+05	1.22E+06	3.05E+06	4.72E+06	2.66E+04	1.50E+07	2.45E+07	5.37E+06	2.19E+07	5.28E+06
palmitate	1.50E+09	3.84E+09	2.31E+09	5.31E+09	1.02E+09	5.90E+09	4.65E+09	2.04E+09	8.06E+06	3.85E+09
palmitoleate	8.95E+07	2.16E+06	8.78E+06	8.69E+07	3.33E+07	2.82E+08	5.07E+07	9.96E+07	1.19E+08	2.98E+08
pendtadenoyl carnitine	5.5	1.0	7.1	4.0	8.4	1.1	3.2	1.9	2.4	6.1

	8E+05	8E+05	2E+05	4E+06	2E+05	4E+06	8E+06	7E+06	1E+06	8E+05
pentadecanoate	7.04E+06	8.62E+06	1.58E+07	5.09E+07	2.91E+07	4.75E+07	3.77E+07	1.10E+07	2.80E+07	3.93E+07
Perillic acid	1.11E+06	6.20E+05	1.32E+06	4.08E+05	1.02E+06	3.74E+06	8.03E+06	2.70E+06	1.61E+06	3.23E+06
perillyl aldehyde	6.83E+05	3.60E+05	5.34E+04	6.64E+05	9.37E+04	2.43E+06	8.08E+06	7.88E+05	4.19E+06	2.70E+06
phenylacetaldehyde	9.56E+04	6.16E+04	1.23E+04	1.81E+05	2.68E+05	1.00E+06	1.39E+06	3.13E+05	3.07E+05	1.75E+05
Phosphodimethylethanolamine	1.48E+05	1.36E+05	1.83E+05	3.35E+05	3.47E+05	2.84E+05	2.90E+05	5.42E+05	7.12E+05	2.97E+05
Phosphoenolpyruvate	2.65E+05	3.30E+04	5.50E+05	4.02E+04	3.81E+05	1.68E+06	5.47E+06	4.11E+05	1.77E+05	9.45E+04
phytanic acid/arachidic acid	1.92E+06	1.69E+06	2.08E+06	1.47E+07	1.05E+06	2.97E+06	3.55E+06	3.02E+05	2.74E+06	1.34E+06
pristanic acid	1.73E+06	5.61E+05	1.27E+06	2.90E+06	2.14E+05	5.86E+06	5.06E+06	3.30E+05	5.97E+05	1.57E+06
propionyl-carnitine	1.74E+07	3.76E+07	7.76E+07	3.20E+07	7.85E+07	5.79E+07	1.13E+07	9.16E+07	4.27E+07	2.47E+06
Pyridoxal	1.20E+06	3.22E+05	1.18E+06	9.68E+05	5.15E+05	2.73E+05	1.13E+06	3.00E+04	1.52E+06	4.41E+05
pyruvate	5.7	8.3	1.1	1.1	9.2	1.9	2.4	3.3	1.4	3.5

	4E+05	4E+06	2E+07	3E+07	4E+06	0E+07	4E+07	1E+06	1E+07	9E+06
Riboflavin	5.22E+05	5.76E+04	7.55E+05	5.08E+05	2.11E+05	7.90E+05	2.33E+06	2.65E+06	9.13E+05	7.79E+04
ribose-phosphate	4.66E+06	1.61E+06	1.68E+06	1.93E+06	5.19E+06	4.93E+07	1.39E+07	1.44E+07	4.08E+07	5.75E+06
S-[2-carboxy-1-(1 H-imidazol-4-yl)ethyl]-L-cysteine	2.40E+05	2.29E+05	1.76E+05	8.43E+05	3.37E+05	1.76E+05	7.21E+03	2.17E+05	2.48E+05	7.36E+05
S-Adenosyl-L-homocysteine	1.94E+06	5.51E+04	8.86E+05	1.47E+06	1.34E+06	1.54E+06	2.75E+05	2.46E+06	6.13E+05	4.46E+05
S-adenosyl-L-methionine	1.84E+06	1.10E+06	1.02E+06	2.42E+06	1.88E+06	6.66E+06	1.46E+07	1.90E+07	2.19E+07	5.15E+06
sarcosine	5.66E+07	8.01E+07	2.35E+07	8.54E+07	8.75E+07	1.97E+08	4.29E+06	1.65E+08	7.03E+07	4.65E+07
Sedoheptulose 7-phosphate	6.95E+05	4.40E+05	1.01E+06	7.18E+05	5.33E+05	8.13E+06	1.29E+07	8.70E+05	1.31E+04	1.88E+06
sn-Glycero-3-phosphocholine	2.05E+09	8.93E+08	2.62E+09	1.60E+09	3.12E+07	4.55E+09	2.65E+09	6.12E+09	7.57E+08	4.73E+08
sphingosine/(2S)-1-hydroxy-3-oxooctadecan-2-aminium	6.29E+05	1.57E+05	1.92E+06	1.64E+07	2.48E+06	5.83E+06	4.83E+05	7.09E+06	7.84E+06	2.58E+06
stearate	3.14E+07	8.67E+08	9.96E+08	3.88E+08	2.65E+08	9.54E+07	6.58E+08	6.65E+08	4.29E+08	7.97E+08
stearoylcarnitine	7.0	3.3	2.1	5.5	4.0	4.1	1.1	4.7	1.5	5.3

	3E+06	8E+06	3E+07	4E+07	9E+07	6E+07	4E+08	6E+07	1E+08	6E+07
succinate(2-)	6.97E+07	7.01E+07	6.19E+07	1.31E+08	1.16E+08	2.18E+08	5.42E+07	1.29E+08	2.76E+08	7.09E+05
succinyl carnitine	5.61E+06	7.61E+06	3.09E+05	4.74E+06	1.52E+05	1.01E+07	2.44E+07	5.25E+06	1.37E+07	1.79E+07
Taurine	1.20E+09	4.29E+08	1.37E+09	1.31E+09	1.22E+09	3.04E+09	1.55E+09	2.19E+09	1.31E+09	1.39E+09
Tetracosatetraenoyl carnitine	8.66E+04	3.73E+04	3.18E+04	3.46E+04	4.46E+04	1.37E+05	5.22E+05	3.79E+05	3.90E+05	3.30E+05
tetradecanoyl carnitine(myristoyl carnitine)	1.51E+07	2.73E+06	1.09E+07	1.42E+07	6.12E+06	5.40E+07	1.24E+08	2.24E+07	4.90E+07	2.20E+07
tetradecenoate (n-C14:1)	2.92E+06	1.62E+06	1.78E+06	2.45E+06	1.88E+06	1.79E+07	1.47E+07	3.41E+05	1.50E+07	5.79E+06
tetradecenoyl carnitine	9.85E+05	3.29E+05	1.51E+05	1.06E+06	3.00E+05	2.48E+05	2.04E+06	3.07E+06	7.47E+05	2.05E+05
thiosulfate	1.63E+06	2.42E+06	1.56E+05	3.26E+06	8.12E+05	1.42E+06	4.77E+06	8.26E+05	1.96E+06	1.22E+06
thymine/imidazol-4-ylacetate	7.93E+05	3.78E+05	1.09E+06	3.48E+06	5.19E+05	1.65E+07	9.68E+06	4.37E+06	7.34E+06	1.57E+06
tiglyl carnitine	1.55E+06	1.04E+05	1.80E+06	1.31E+06	2.05E+06	2.64E+06	1.53E+06	2.04E+06	1.99E+05	2.23E+06
trans-4,5-epoxy-2(E)-decenal	3.6	1.1	2.2	2.9	1.4	1.5	3.6	1.2	1.9	3.6

	9E+05	1E+05	6E+05	0E+05	2E+05	5E+06	8E+06	3E+06	1E+06	5E+05
trans-Hexadec-2-enoyl carnitine	6.18E+06	1.09E+07	3.22E+06	2.23E+07	2.67E+06	4.18E+06	4.26E+07	5.05E+07	5.71E+07	4.05E+07
Trimethylamine N-oxide	3.71E+05	2.12E+05	1.59E+06	1.53E+05	1.24E+06	3.66E+05	1.47E+06	2.71E+05	5.79E+05	1.96E+06
UDP-D-glucose/UDP-D-galactose	5.19E+06	5.36E+06	7.10E+06	1.58E+06	4.04E+06	2.80E+07	1.17E+07	1.98E+07	3.35E+07	2.76E+07
UDP-D-glucuronate	1.22E+06	5.99E+05	1.99E+06	7.87E+04	3.95E+05	6.79E+06	1.66E+05	2.62E+06	7.55E+05	1.26E+06
UDP-N-acetyl-alpha-D-glucosamine/UDP-N-acetyl-alpha-D-galactosamine	1.67E+07	7.60E+06	2.87E+07	2.21E+07	2.70E+07	1.58E+08	1.18E+08	9.39E+07	4.57E+08	2.27E+08
uracil	5.69E+06	1.63E+07	1.93E+07	3.11E+05	1.18E+07	8.91E+07	2.45E+07	1.78E+07	1.77E+07	3.46E+07
Ureidopropionic acid/Glycyl-glycine	3.25E+07	3.87E+06	3.89E+06	1.34E+07	7.11E+06	2.55E+07	7.17E+07	2.91E+07	4.82E+07	3.04E+07
Urocanate	1.56E+07	2.15E+07	1.21E+08	5.75E+08	2.51E+07	2.11E+08	1.28E+08	6.26E+07	8.86E+07	4.46E+06
Valine	6.02E+07	6.56E+06	4.07E+07	3.44E+07	5.67E+07	1.22E+08	3.73E+07	9.06E+07	4.93E+07	4.98E+07
w-hydroxydecanoicacid	2.63E+06	2.09E+06	3.62E+05	6.04E+05	2.85E+05	6.50E+06	1.75E+07	1.04E+07	9.96E+06	6.73E+05
Xanthine	2.5	4.3	7.5	9.8	1.1	8.5	2.2	4.4	1.2	5.4

	5E+06	7E+06	0E+06	7E+07	5E+07	8E+07	8E+08	6E+07	2E+08	3E+07
xanthosine	5.18E+04	3.44E+04	1.49E+05	3.69E+05	7.14E+04	7.73E+03	2.56E+05	8.90E+03	1.85E+04	3.93E+04

Table S4. 214 genes were identified in both DEGs with different intervention groups.

GM43920
GOT2-PS1
IFIT1
GM18537
SPEF2
AC122821.1
GM2541
GM19510
GM14401
GM48990
SEC23A
5430403G16RIK
GM13136
TARBP2
RCE1
GM11273
EMC10
SNRNP35
ZFP703
TLNRD1
INO80B
SPNS1
FBXL19
GM11808
44805
GM14681
FOXP4
TSPAN4
PLPP2
OGFOD2
SRF
KLF16
RPS28

HMGA1B
DUSP8
CLDN3
RABAC1
HNRNPA0
RAI1
ARMC5
TTYH3
GM10443
6330403L08RIK
CDH24
MIEN1
H1FX
ESRRA
EPN1
SPHK1
DUS3L
ATF5
GM11703
SLC35E4
GM3788
RRP9
ATP6V0C
LINGO1
FAM219A
ATF6B
ADAMTSL5
1810032O08RIK
CLCF1
RPP25L
TMEM259
TOMM40L
CAPN15
NRADD
PSD
TSPO
MAP3K10
ZMYND15
IBA57
LEMD2
MAZ
H2AFX
GM12960

MFSD10
BC037034
KCNC3
NR2F6
RPS10-PS1
PPP2R3D
SSBP4
GM15682
GM8129
ZFP771
OPLAH
PTMS
GM6485
1600002K03RIK
JUND
ZFP628
ALDH16A1
APOC4
JUNB
GM13192
PEX14
DDAH2
KLHDC8B
CRB3
PKMYT1
BC029722
GNAS
GM13123
TMEM205
IER5L
ASIC1
ERF
TNFRSF12A
PRR7
GM21887
NDUFA12
GM45133
GM15247
GM10177
H2-BL
SSSCA1
RPS18-PS3
GM10335

BBC3
GM10132
TAF1C
TMEM238
MIR5125
LARS2
TMEM198
RASL10B
RPL7A-PS5
CRLF2
ABCG4
RAC3
NDUFAF8
GADD45GIP1
1700020L24RIK
CHAC1
MRPS34
GM14321
GM10736
1110065P20RIK
RHBDD3
3010003L21RIK
SH3BP1
GM49322
RNF26
TMEM160
HS3ST6
DOHH
SPATA5L1
FBXL15
NPFF
SNORD49A
CT010467.1
GDF15
CAMK2N2
LRFN1
ANGPTL8
RHBDL1
TTLL11
RPL28-PS3
NRTN
GM26917
RPL23A

GM3756
FAM131C
GM10263
RTN4RL2
SCAND1
GM17111
CAAA01118383.1
ATP5K
PDXP
6330562C20RIK
NKPD1
GM47283
SLC39A5
GM9908
PTP4A1
SEMA6C
B330016D10RIK
AMN
GM14685
COX20
GM11702
GM43555
SDHAF1
GM20949
GM18194
CAAA01147332.1
SNORD32A
GSTP2
GM10709
GM10138
SNORA78
GM28529
GM8210
GM7774
GM9523
PRR22
VWA1
GM16174
GM30025
GM44349
KCNH3
GM23935
4930413G21RIK

KCNIP2
SNORA2B
MIR142B
MIR23A
GM48699
CPT1B
GM11767
HSD11B2
MRC2
